# Influence of bariatric surgery on the peripheral blood immune system of female patients with morbid obesity revealed by high-dimensional mass cytometry

**DOI:** 10.3389/fimmu.2023.1131893

**Published:** 2023-05-11

**Authors:** Adrian Gihring, Fabian Gärtner, Laura Mayer, Aileen Roth, Hend Abdelrasoul, Marko Kornmann, Leonard Elad, Uwe Knippschild

**Affiliations:** Department of General and Visceral Surgery, Surgery Center, Ulm University Medical Center, Ulm, Germany

**Keywords:** obesity, bariatric surgery, inflammation, mass cytometry (CyTOF), immune cells

## Abstract

**Introduction:**

Obesity is associated with low-grade chronic inflammation, altered levels of adipocytokines, and impaired regulation of gastrointestinal hormones. Secreted, these factors exert immunostimulatory functions directly influencing peripheral immune cells.

**Methods:**

In the realm of this study, we aimed to investigate the composition and activation status of peripheral blood immune cells in female patients with morbid obesity compared to lean controls using high-dimensional mass cytometry. Besides, we also assessed the influence of bariatric surgery with respect to its ability to reverse obesity-associated alterations within the first-year post-surgery.

**Results:**

Patients with morbid obesity showed typical signs of chronic inflammation characterized by increased levels of CRP and fibrinogen. Apart from that, metabolic alterations were characterized by increased levels of leptin and resistin as well as decreased levels of adiponectin and ghrelin compared to the healthy control population. All these however, except for ghrelin levels, rapidly normalized after surgery with regard to control levels. Furthermore, we found an increased population of monocytic CD14^+^, HLA-DR^-^, CD11b^+^, CXCR3^+^ cells in patients with morbid obesity and an overall reduction of the HLA-DR monocytic expression compared to the control population. Although CD14^+^, HLA-DR^-^, CD11b^+^, CXCR3^+^ decreased after surgery, HLA-DR expression did not recover within 9 – 11 months post-surgery. Moreover, compared to the control population, patients with morbid obesity showed a perturbed CD4+ T cell compartment, characterized by a strongly elevated CD127^+^ memory T cell subset and decreased naïve T cells, which was not recovered within 9 – 11 months post-surgery. Although NK cells showed an activated phenotype, they were numerically lower in patients with morbid obesity when compared to healthy controls. The NK cell population further decreased after surgery and did not recover quantitatively within the study period.

**Conclusions:**

Our results clearly demonstrate that the rapid adaptions in inflammatory parameters and adipocytokine levels that occur within the first year post-surgery do not translate to the peripheral immune cells. Apart from that, we described highly affected, distinct immune cell subsets, defined as CD127^+^ memory T cells and monocytic CD14^+^, HLA-DR, CD11b^+^, CXCR3^+^ cells, that might play a significant role in understanding and further decoding the etiopathogenesis of morbid obesity.

## 1 Introduction

Obesity was firstly described as a disease by the World Health Organization in the year 2000 thanks to its significantly growing prevalence and serious threat to public health (1). The most severe form of obesity, referred to as morbid obesity, is defined by a body mass index (BMI) of ≥ 35 kg/m^2^ accompanied by one or more severe comorbidities or a BMI of ≥ 40 kg/m^2^ (2). Although obesity is on a rise in all age groups, morbid obesity shows the same or even a higher growth rate compared to milder forms of obesity (3). It’s worth noting, that obesity in patients with a BMI between 55 and 55.9 kg/m^2^ is associated with a reduction in life expectancy of up to 13.7 years (4). Morbid obesity is known to be directly or indirectly associated with as many as 60 clinically relevant ailments. Among them are type 2 diabetes (DM2), different kinds of malignancies (breast, colon, etc.), gallbladder disease, sleep apnea, respiratory problems, and osteoarthritis (5). Furthermore, obesity is an elevated risk factor for cardiovascular diseases like coronary heart disease due to hypertension, dyslipidemia and hyperinsulinemia.

Most of these diseases are sequelae of severe metabolic alterations evoked by an obesity-associated dysfunction of adipose tissue leading to a chronic low-grade inflammation, persistent in patients with morbid obesity. Pathological increase of adipose tissue affects number and qualitative functioning of adipose tissue-resident immune cells leading to an altered secretion profile of pro- and anti-inflammatory cytokines as well as adipocytokines including leptin, adiponectin and resistin (6, 7). Apart from that, gastrointestinal hormones like ghrelin and glucose-dependent insulinotropic polypeptide (GIP), involved in regulation of energy homeostasis, insulin secretion, and appetide control, have been shown to be dysregulated in obesity (8). Since adipocytokines and gastrointestinal hormones are released into the circulatory system, where immune cells express the respective receptors, the immune-stimulatory or -inhibitory function of these hormones (9) might strongly be involved in regulating the chronic inflammation observed in obesity.

The chronic inflammation in patients with morbid obesity is hypothesized to highly influence the immune system resulting in an increased susceptibility for infections (10) and cancer (11). Different peripheral blood immune cell subsets including CD4+ T cells, B cells, NK cells, as well as monocytes have been shown to be highly influenced by morbid obesity (12). The T cell compartment is perturbed as a result of a thymic dysfunction characterized by an accumulation of memory T cells and decreased naïve T cells (13). NK cells undergo obesity-induced metabolic reprogramming limiting their function (14), whereas monocytes are primed towards a pro-inflammatory phenotype and concomitantly accumulate immune-suppressive subsets (15).

Bariatric surgery, exemplified by sleeve gastrectomy and proximal gastric bypass surgeries, is the most effective and sustainable treatment for weight loss, relief of obesity-associated comorbidities like DM2 and resolution of chronic inflammation (16–18). Although bariatric surgery seems to highly improve the quality of life (19), long-term side-effects like malnutrition or an increased risk of anemia have been observed (20). The effect of bariatric surgery on the immune system is still not fully elucidated. Several studies observed positive adaptions of immune cell populations after bariatric surgery including NK cells, monocytes, B cells, and T cells (2, 21–23). However, these studies often focused on a chosen few immune cell populations or just referred to the cell count without a detailed analysis of the immune cell populations phenotyping.

Therefore, the aim of this study was to perform high-resolution phenotyping of immune cell populations detectable in whole blood samples of patients with morbid obesity and lean controls using mass cytometry, and to study the effect of bariatric surgery on these immune cell populations. We detected increased populations of CD127^+^ CD4+ memory T cells and monocytic CD14^+^, HLA-DR^-^, CD11b^+^, CXCR3^+^ cells as well as decreased levels of naïve CD4+ T cells and NK cells; a phenomenon, which did not fully reverse within 11 months after surgery. These results give further insight into the impact of bariatric surgery on the already impaired immune system of patients with morbid obesity and highlight the importance of a careful monitoring after bariatric surgery to prevent functional restrictions in a long-term perspective.

## 2 Material and methods

### 2.1 Ethics

The study protocol was approved by the ethics committee at Ulm University (ethical grant no. 30-20). The protocol was conducted following the Declaration of Helsinki. All study participants were extensively informed by an attending physician and gave their written, explicit consent. Data of study participants was pseudonymized.

### 2.2 Study cohorts and sampling

Female, adult (≥ 18 years) patients that underwent bariatric surgery at Ulm University Hospital between 2020 and 2022 and met the criteria according to the “German S3-guideline: Surgery for obesity and metabolic diseases” (BMI ≥ 40 kg/m^2^ or BMI ≥ 35 kg/m^2^ with one or more obesity-associated comorbidities) were included in the study. Patients suffering from inflammatory bowel disease, systemic inflammatory disease, acute infections, cancer, autoimmune disease or receiving immunosuppressive therapy were excluded from the study. Study participants included in the patient group were of non-Hispanic White ethnicity.

Initial baseline comparison (prior to surgery) using conventional flow cytometry included 38 female patients and 10 healthy, female age-matched controls (CTRL). The CTRL group consisted of non-Hispanic White normal weight (BMI ≥ 18.5 and BMI ≤ 25.0 kg/m^2^), female employees engaged at Ulm University Hospital. According to a self-declaration of health, study participants of the CTRL group did not suffer from any acute and chronic diseases or physical restrictions. Furthermore, study participants of the CTRL group did not receive medications including antihypertensives, antidepressants, antidiabetics, statins, vitamin supplements, thyroid hormones, proton-pump inhibitors, or anti-inflammatory drugs. Additionally, clinical blood parameters were determined for the CTRL cohort to exclude inflammatory and metabolic disorders (**Supplementary Table 1**).

To assess the effect of bariatric surgery on the peripheral blood immune system, a subcohort of 12 female patients was randomly selected and samples were acquired at baseline, 1 – 2 months post-surgery (p.s.), 3 - 5 months p.s., 6 - 8 months p.s. and 9 - 11 months p.s. using high-dimensional mass cytometry by time of flight (CyTOF), whole-blood-based quantitative reverse transcription polymerase chain reaction (qRT-PCR) and bead-based immunoassays. The previously described control cohort was used as reference. Four weeks prior to surgery, patients were on a strict diet consisting of 40% carbohydrates, 25% protein and 35% fat as well as a daily minimum of 30 g dietary fibers. The aim of the diet was to reduce liver size making surgery more feasible and consequently minimizing complications.

EDTA-blood samples were collected one day prior to surgery and at the respective time-points within follow-up examinations at Ulm University Hospital and subsequently processed in the laboratory. Patients and controls were not in a controlled fasted state at blood draw.

### 2.3 Anthropometric measurements and clinical data

The BMI in kg/m^2^ describes the ratio of the person’s weight in kilograms to the squared height in meters. Body weight and body height were determined prior to surgery and at the respective time-points during follow-up examinations at Ulm University Hospital. Blood samples were taken routinely and analyzed in the Department of Clinical Chemistry at Ulm University Hospital (accredited according to DIN EN ISO 15189). Reference values were also obtained from the Department of Clinical Chemistry at Ulm University Hospital.

### 2.4 Flow cytometry staining and analysis

Peripheral blood mononuclear cells (PBMCs) were isolated from 3 mL EDTA-blood using Ficoll-Paque™ PLUS (Cytiva). Blood was mixed with equal volume of PBS (Gibco), layered over Ficoll and centrifuged for 20 min and 300 x g at room temperature (RT) with breaks off. PBMCs were aspirated and washed twice with 10 mL PBS and once with 1 mL staining buffer (1 x PBS, 1% BSA, 2 mM EDTA, 0.05% NaN_3_) each time followed by a centrifugation step (300 x g, 8 min, RT). Staining of 1.0 x 10^6^ cells was performed in the dark for 30 min at 4°C using 100 µL staining buffer containing fluorescent labelled antibodies (dilution 1:100) specific to CD3 (VioBlue, Miltenyi Biotec, 130-114-519), CD4 (PE, Miltenyi Biotec, 130-113-225), CD8 (APC, Miltenyi Biotec, 130-110-679), CD19 (FITC, Miltenyi Biotec, 130-113-645), CD56 (PE-Vio 770, Miltenyi Biotec, 130-113-313), CD14 (APC-Vio 770, Miltenyi Biotec, 130-110-522), CD16 (VioGreen, Miltenyi Biotec, 130-113-397) and HLA-DR (PerCP-Vio 700, Miltenyi Biotec, 130-111-793). Cells were washed with 2 mL staining buffer, centrifuged (300 x g, 8 min, RT), resuspended in 500 µL staining buffer and subsequently acquired on a MACSQuant^®^ Analyzer 10 Flow Cytometer (Miltenyi Biotech). Compensation was performed using instrument-specific automated compensation with single-stained compensation beads. To exclude background staining and unspecific binding of antibodies, isotype controls have been measured. All samples were acquired with the same voltage settings. Analysis of data was performed using Flowlogic™ 8.4 software (Inivai Technologies) according to the provided gating scheme (**Supplementary Figure 1**). To analyze and exclude age-related effects on major immune cell subsets, patient cohort was subdivided into two age groups (20 – 45 years and 45 – 61 years) (**Supplementary Figure 2**).

### 2.5 Mass cytometry staining

One mL of EDTA-blood was mixed with 1 mL SmartTube Proteomic Stabilizer PROT1 (SMART TUBE Inc.), incubated for 10 min at RT and stored at -80°C. Samples were thawed and erythrocyte lysis was performed using 1x Thaw-Lysis buffer (SMART TUBE Inc.) according to manufacturer’s specifications. After lysis, cells were resuspended in 2 mL CyFACS buffer (1 x PBS (Rockland, MB-008), 1% BSA, 2 mM EDTA, 0.05% NaN_3_) and cell concentration was determined. A total number of 1.5 x 10^6^ cells per sample were used for the staining procedure. One mL CyFACS buffer was added, cells were centrifuged (600 x g, 8 min, RT) and supernatant was discarded. To prevent unspecific antibody staining, Fc receptor (FcR) block was performed by adding 3 µL of human FcR Blocking Reagent (Miltenyi) to the cells followed by an incubation for 20 min at RT. Master mix containing the antibodies listed in **Supplementary Table 2** was prepared shortly prior to staining and filtered with 0.1 µM spin filter units (Merck).

One hundred µL of master mix was added to each sample and incubated for 30 min at 4°C. Titration of antibody panel was performed prior to the first experiments to determine the antibody concentration. Antibodies that were not purchased from Fluidigm were conjugated in-house using the respective Maxpar X8 Antibody Labeling Kit (Fluidigm) according to manufacturer’s specifications.

Cells were washed two times with 1 mL CyFACS buffer (600 x g, 8 min, RT). Fixation was performed by adding 1 mL of 3% methanol-free paraformaldehyde (PFA) (Thermo Scientific Pierce) in CyPBS followed by an incubation for 2 h at 4°C. Samples were washed twice with 1 mL CyPBS (600 x g, 8 min, RT). Cells were incubated with 0.5 mL of 0.2% Cell-ID Intercalator-Ir (Fluidigm) in Maxpar Fix and Perm Buffer (Fluidigm) for 20 min at RT followed by two washes with 2 mL CyPBS (600 x g, 8 min, RT). Cells were frozen in 1 mL cold freezing medium (10% DMSO in fetal calf serum) and stored at -80°C until day of acquisition.

On the day of acquisition, samples were thawed in a cold-water bath and washed once with 1 mL CyFACS containing Benzonase/Nuclease (Sigma Aldrich, 1:10,000), once with 1 mL CyFACS without Benzonase and three times with Maxpar Water (Fluidigm) each time centrifuged at 600 x g for 8 min at RT. Samples were adapted to a cell concentration of 10^6^ cells/mL and 300,000 cells were measured at 300 events/s on a Helios system (Fluidigm). EQ Four Element Calibration Beads (Fluidigm) were used for normalization over time.

### 2.6 Treatment of anchor samples

Anchor samples consisting of buffy-coat derived PBMCs (purchased from Institute for Clinical Transfusion Medicine and Immunogenetics (IKT), Ulm, Germany) were adapted to a concentration of 1.0 x 10^6^ cells/mL. One mL aliquots were mixed with 1 mL SmartTube Proteomic Stabilizer PROT1 (SMART TUBE Inc.) and frozen at -80°C. Anchor samples were treated the same way as patient samples and one anchor was included in each batch of measured patient samples.

### 2.7 Mass cytometry data acquisition and analysis

Generated raw FCS files were preprocessed prior to normalization (**Supplementary Figure 2A**). Calibration beads were removed, and DNA positive cells were identified (^191^Ir and ^193^Ir). Doublets were excluded using gaussian parameters event length and residual (24). Batch normalization was performed using R-based CyTOF Batch Adjust workflow (25) adapted for Windows system. Channel specific batch-to-batch variation was evaluated using anchor samples and adjustment factors were transferred to patient samples (**Supplementary Figure 2B**).

After batch normalization, FCS files were uploaded to Cytobank (Beckman Coulter), transformed (arcsinh, co-factor = 5) and analyzed using manual gating as well as machine learning algorithms including dimensionality reduction *via* Uniform Manifold Approximation and Projection (UMAP) (26) and clustering *via* Self-Organizing Map (SOM) algorithm FlowSOM (27). Twenty thousand cells per sample were included in the automated analysis. For the illustration of the UMAP density-plots, FCS files were exported and concatenated according to their corresponding group using the CATALYSTLite online application (28).

In order to analyze populations of interest at a higher resolution, manually gated populations were exported from Cytobank as raw value FCS files. Validation of the gating was performed by comparing percentages of the manually gated populations with the percentages of populations identified with FlowSOM using linear regression analysis (**Supplementary Figure 3**). For monocytes, manual gating seemed to be prone to slight underestimation of the population. This might be due to high heterogeneity within the monocyte population that could be better depicted with the algorithm (29). Mean frequencies of manually gated CD45^+^ cells can be found in the supplement (**Supplementary Table 3**).

For the detailed analysis of specific cell populations the Spectre R package was used (30) with instructions and source code provided at https://github.com/ImmuneDynamics/spectre. The validated population of interest was exported from Cytobank as raw value FCS files. The arcsinh transformed (co-factor 5) dataset was merged into a single data.table, with keywords denoting the sample, group, and other factors added to each row (cell). The FlowSOM algorithm (27) was then run on the merged dataset to cluster the data, where every cell is assigned to a specific cluster and metacluster. Subsequently, the data was randomly downsampled to 10,000 cells per group and analyzed by the dimensionality reduction algorithm UMAP (26) for cellular visualization. Annotated clusters were depicted in a heatmap as normalized abundances with respect to the mean of the CTRL population. Statistical analysis was performed as described in the corresponding chapter “statistical analysis”.

Marker expression of clusters was shown as volcano plots generated with an adapted version of the EnhancedVolcano R script (31). Marker expression of each analyzed surface marker is shown as log_2_ fold change (FC) calculated based on the cluster-specific mean fluorescence intensities (MFIs) of the respective groups. log_2_ FC (x-axis) is plotted against the statistical significance (y-axis) shown as -log_10_ p. Statistical significance was calculated based on cluster-specific MFIs using an unpaired two-sided Wilcoxon test (α = 0.05).

A list of the immune cell populations that have been identified via mass cytometry as well as the corresponding surface markers that have been used for identification is shown in the supplement (**Supplementary Table 4**).

The R scripts used for the analysis of the data are available as PDF documents in the supplement.

### 2.8 RNA isolation

Two hundred µL of EDTA-blood were mixed with equal volumes of Monarch^®^ DNA/RNA Protection Reagent (New England BioLabs), vortexed and stored at -80°C. RNA isolation was performed according to manufacturer’s specifications. Samples were thawed at RT and 10 µL proteinase K were added. Samples were vortexed and incubated for 30 min at RT. Four hundred µL isopropanol were added, vortexed, transferred to an RNA purification column and centrifuged (16,000 x g, 30 sec). For removal of residual genomic DNA on-column DNAse I treatment was performed. Priming buffer (0.5 mL) was added followed by two wash steps with RNA Wash Buffer. RNA was eluted with 50 µL nuclease-free water. RNA concentration was determined using QIAxpert System.

### 2.9 qRT-PCR analysis

One hundred and fifty ng total RNA were transcribed into complementary DNA (cDNA) using the AffinityScript Multiple Temperature cDNA Synthesis Kit (Agilent Technologies) with oligo(dT) primers according to manufacturer’s specifications. Samples were adjusted to a final concentration of 1 ng/µL with nuclease-free water. Prior to qRT-PCR, samples were tested for genomic DNA (gDNA) contamination using exon-specific primers (Forward: 5’-TCT GCC GTT TTC CGT AGG ACT CTC-3’, Reverse: 5’-CCC TGG ATG TGA CAG CTC CCC-3’) and cDNA functionality using intron-specific primers (Forward: 5’-GGC ATC CTC ACC CTG AAG TA-3’, Reverse: 5’- GTC AGG CAG CTC GTA GCT CT-3’) for *ACTB* in conventional PCR. After exclusion of gDNA contamination and confirmation of cDNA functionality, qRT-PCR was performed using QuantiTect SYBR Green PCR kit (Qiagen) and QuantiTect Primer assays specific for *GAPDH* (QT00273322), *RPLP0* (QT00075012), *LEPR* (QT00006524), *ADIPOR1* (QT00002352), *GIPR* (QT00033138) and *GHRL* (QT00041377). *GAPDH* and *RPLP0* were validated and used as housekeeping genes (**Supplementary Figure 4**). Melting curves of PCR products were evaluated and should have been between 72°C to 86°C according to manufacturer’s specifications. Calculated ΔCT values of patients were normalized to the mean ΔCT value of control population resulting in depicted x-fold values.

### 2.10 Bead-based immunoassay

EDTA-blood was centrifuged at 2,600 x g for 15 min and plasma was stored at -80°C. Samples were thawed and analyzed in duplicates using the LEGENDplex Human Metabolic Panel with a V-bottom plate (BioLegend). The measured analytes included adiponectin (beads A4), adipsin (beads A5), leptin (beads B4) and resistin (beads B7). Staining and set-up were performed according to the manufacturer’s specifications. Data was acquired on MACSQuant^®^ Analyzer 10 Flow Cytometer (Miltenyi Biotech) and analysis was performed with the Legendplex Cloud-based software (BioLegend, Version 2022-07-15).

### 2.11 Enzyme-linked immunosorbent assay

Enzyme-linked Immunosorbent Assays (ELISAs) were performed with plasma samples using commercially available ELISA kits for Ghrelin (ThermoFisher, # BMS2192) and GIP (Merck, EZHGIP-54K) according to the manufacturer’s specifications. Absorption was determined at 450 nm in Tecan Spark^®^ Multimode Reader.

### 2.12 Statistical analysis

GraphPad Prism 7.04 (GraphPad Software, Inc.) was used for statistical analysis. The specific tests performed are described below each graph. When comparing two groups, a two-tailed Student’s *t* test (α = 0.05) was performed if requirements of normal distribution (Shapiro-Wilk test) and homogeneity of variances (F-test) were given. Otherwise, a Welch’s test or a Mann-Whitney test were performed. When comparing more than two groups, a one-way ANOVA with Geisser-Greenhouse correction followed by an uncorrected Fisher’s LSD test (α = 0.05) was performed when normal distribution (Shapiro-Wilk test) was given. Otherwise, a Kruskal-Wallis test with Geisser-Greenhouse correction followed by an uncorrected Dunn’s test was performed. The specific method is stated in the figure legend. For each experiment, the sample sizes are depicted in the figure or in the figure legend. The following indicators were used for all statistical tests: * indicates p < 0.05, ** indicates p < 0.01, *** indicates p < 0.001, and **** indicates p < 0.0001. Data is depicted either as boxplots showing full range or as heatmaps showing either row z-score normalized data or min-max scaled data (surface marker expression), unless otherwise stated.

## 3 Results

### 3.1 Low-grade chronic inflammation was prevalent in the main cohort of female patients with morbid obesity prior to bariatric surgery

The health status of 38 female patients with morbid obesity who underwent bariatric surgery was assessed by determining pre-existing comorbidities, blood pressure, medication, as well as several blood parameters (**Table 1**). The median BMI of the cohort was 48.9 kg/m^2^. The predominant procedure performed was sleeve gastrectomy (79%) followed by gastric bypass (21%). Patients with morbid obesity frequently suffered from diabetes and its preliminary stages (66%) and hyperlipoproteinemia (39%), which is also reflected in the blood values. Considering the measured blood parameters, patients showed typical obesity-associated signs of a low-grade chronic inflammation characterized by significantly elevated levels of both circulating C-reactive protein (CRP) (median= 7.7 mg/L) and circulating fibrinogen (median = 3.7 g/L) (32). The blood fat values for cholesterol (11 patients ≥ 5 mmol/L), triglycerides (13 patients ≥ 1.7), and low-density lipoprotein (LDL) (12 patients ≥ 3 mmol/L) were shifted towards the upper limit of normal levels, whereas the value of high-density lipoprotein (HDL) (11 patients ≤ 1.2 mmol/L) was shifted towards the lower limit of normal levels.

**Table 1:** Characteristics of female baseline cohort (n = 38) including age, body mass index (BMI), procedure, comorbidities, blood pressure, medication, and blood parameters.

Female baseline cohortn = 38					
Characteristics		Median [Min. – Max.]			
Age		43.5 [20 – 64]			
BMI [kg/m^2^]		48.9 [37.9 – 64.9]			
Procedure		∑ (%)			
Sleeve gastrectomy		30 (79)			
Gastric bypass (Roux-en-Y)		8 (21)			
Comorbidities		∑ (%)			
Diabetes and preliminary stages		25 (66)			
Arterial hypertension		17 (44)			
Hypothyroidism		13 (34)			
Obstructive sleep apnea		22 (58)			
Depressive disorder		12 (32)			
Hyperlipoproteinemia		15 (39)			
Blood pressure		Median [Min. – Max.]			
Systolic [mmHg]		146.5 [117 – 187]			
Diastolic [mmHg]		87.5 [72 – 138]			
Medication		∑ (%)			
Antidiabetics		21 [55]			
Antihypertensives		17 [44]			
Antidepressants		10 [26]			
Thyroid hormones		14 [37]			
Statins		5 [13]			
Vitamin supplements		9 [24]			
Proton pump inhibitors		8 [21]			
Blood parameters					
Parameter [Unit]	Median [Q1 – Q3]	Min. reference value	Max. reference value	n	p-value
CRP [mg/L]	7.7 [5.7 – 12.4]	–	5	37	<0.0001
Fibrinogen [g/L]	3.7 [3.4 – 4.6]	1.8	3.5	30	0.014
Triglycerides [mmol/L]	1.6 [1.2 – 2.0]	–	1.7	30	ns
Cholesterol [mmol/L]	4.5 [4.0 – 5.6]	–	5	30	ns
HDL [mmol/L]	1.3 [1.2 – 1.6]	1.2	–	30	ns
LDL [mmol/L]	2.7 [2.2 – 3.6]	–	3	30	ns
AST [U/L]	23.5 [21.0 – 30.8]	–	35	32	ns
ALT [U/L]	24.0 [19.0 – 33.0]	–	35	35	ns
Uric acid [µmol/L]	327.0 [267.3 – 356.3]	137	363	30	ns
Creatinine [µmol/L]	66.0 [54.5 – 70.5]	44	80	37	ns
GGT [U/L]	26.0 [18.0 – 32.0]	–	40	31	ns
AP [U/L]	78.5 [66.0 – 90.3]	35	105	32	ns
CK [U/L]	100.0 [70.3 – 128.8]	20	180	30	ns
LDH [U/L]	201.0 [182.0 – 228.5]	–	250	29	ns
Lipase [U/L]	29.0 [21.0 – 33.0]	13	60	31	ns
Albumin [g/L]	43.0 [42.0 – 46.0]	35	53	30	ns
Protein [g/L]	75.0 [72.0 – 78.0]	66	83	31	ns
Quick [%]	102.0 [93.0 – 112.0]	70	130	37	ns
PTT [s]	28.3 [26.8 – 30.0]	26	36	37	ns
Insulin [mU/L]	22.4 [17.1 – 43.3]	2.6	24.9	31	ns
HbA1c (IFCC) [mmol/mol]	37.0 [34.0 – 42.0]	20	42	30	ns
Leukocytes [Giga/L]	7.7 [7.3 – 9.2]	4.4	11.3	38	ns
Erythrocytes [Tera/L]	4.7 [4.4 – 4.9]	4.5	5.9	38	ns
Hemoglobin [g/dL]	13.4 [12.7 – 14.3]	12.3	15.3	38	ns
Hematocrit [L/L]	0.40 [0.38 – 0.42]	0.36	0.45	38	ns
MCV [fL]	85.5 [82.4 – 88.4]	80	96	38	ns
MCH [pg]	28.7 [27.7 – 29.9]	27.5	33.2	38	ns
MCHC [g/dL]	33.6 [33.3 – 34.2]	33.4	35.5	38	ns
RDW [%]	13.8 [13.3 – 14.9]	–	15	38	ns
Thrombocytes [Giga/L]	278.0 [232.5 – 329.3]	150	450	38	ns
MTV [fL]	9.5 [8.6 – 10.1]	6.8	10	38	ns

Statistical analysis was performed using a non-parametric, one-sample Wilcoxon Signed Rank test, designed to compare the mean of the baseline cohort to its respective Min. or Max. reference value using α = 0.05; ns, non-significant. The reference values were obtained from the central institution of clinical chemistry at Ulm University Hospital.

**Figure 1:**
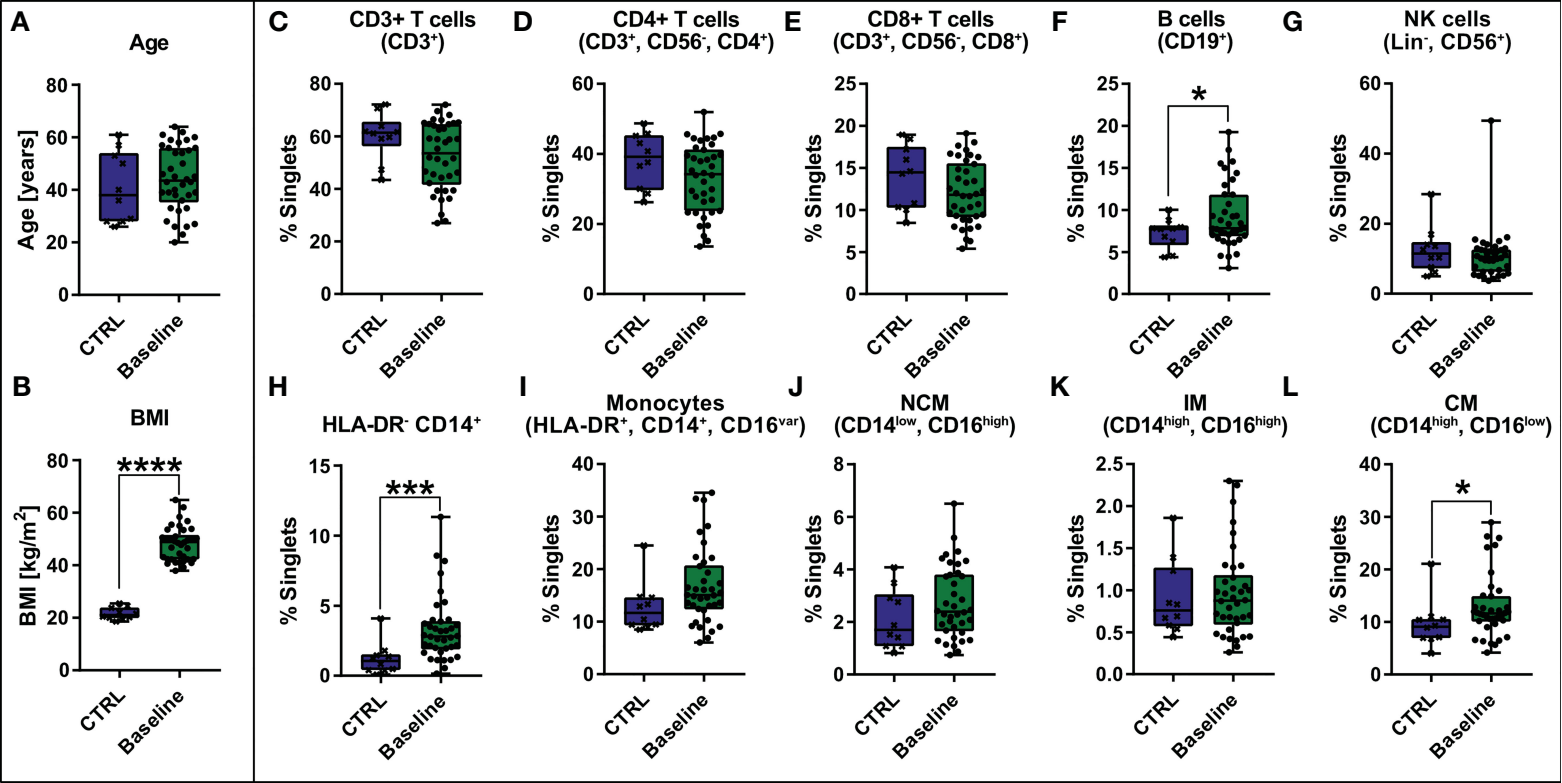
Comparison of age [years] **(A)** and BMI [kg/m^2^] **(B)** between control cohort (CTRL, n = 10) and baseline cohort with morbid obesity (Baseline, n = 38) and comparison of abundance of PBMCs [% singlets] including CD3+ T cells (CD3^+^), CD4+ T cells (CD3^+^, CD56^-^, CD4^+^), CD8+ T cells (CD3^+^, CD56^-^, CD8^+^), B cells (CD19^+^), NK cells (Lin^-^, CD56^+^), HLA-DR^-^ CD14^+^, Monocytes (HLA-DR^+^, CD14^+^, CD16^var^) divided in non-classical monocytes (NCM)(CD14^low^, CD16^high^), intermediate monocytes (IM) (CD14^high^, CD16^high^) and classical monocytes (CM) (CD14^high^, CD16^low^) **(C–L)**. Statistical analysis was performed using a two-sided unpaired t-test (Age, CD3+ T cells, CD4+ T cells, CD8+ T cells, NCM, IM and CM), a Welch’s test (BMI, B cells) or a Mann-Whitney test (HLA-DR^-^ CD14^+^, Monocytes, NK cells) with α = 0.05. *p-value ≤ 0.05, ***p-value ≤ 0.001, ****p-value ≤ 0.0001.

Apart from clinical blood parameters, PBMCs were characterized using flow cytometry. A control cohort (CTRL) consisting of 10 healthy, lean (BMI ≤ 25 kg/m^2^), age- and gender-matched volunteers (**Figures 1A, B**) was established serving as a reference for the patient group with morbid obesity (Baseline). T cells (CD3^+^) (**Figure 1C**) including CD4+ T helper cells (**Figure 1D**) and CD8+ cytotoxic T cells (**Figure 1E**) as well as NK cells (Lin^-^, CD56^+^) (**Figure 1G**) tend to be slightly decreased in patients with morbid obesity. However, statistically these shifts were not significant due to high variation within the cohorts. B cells (CD19^+^) (**Figure 1F**) and monocytes (HLA-DR^+^, CD14^+^, CD16^var^) (**Figure 1I**) tend to be increased in patients with morbid obesity. Within the monocyte compartment the HLA-DR^-^, CD14^+^ cells (**Figure 1H**), referred to as immunosuppressive myeloid-derived suppressor cells (MDSCs) (15, 33), and the classical monocytes (CM) (**Figure 1L**) were significantly increased in patients with morbid obesity, whereas no difference was observed for intermediate monocytes (IM) (**Figure 1K**) and non-classical monocytes (NCM) (**Figure 1J**).

### 3.2 Bariatric surgery led to significant weight loss and improved inflammatory and metabolic blood parameters in a female sub cohort

**Figure 2:**
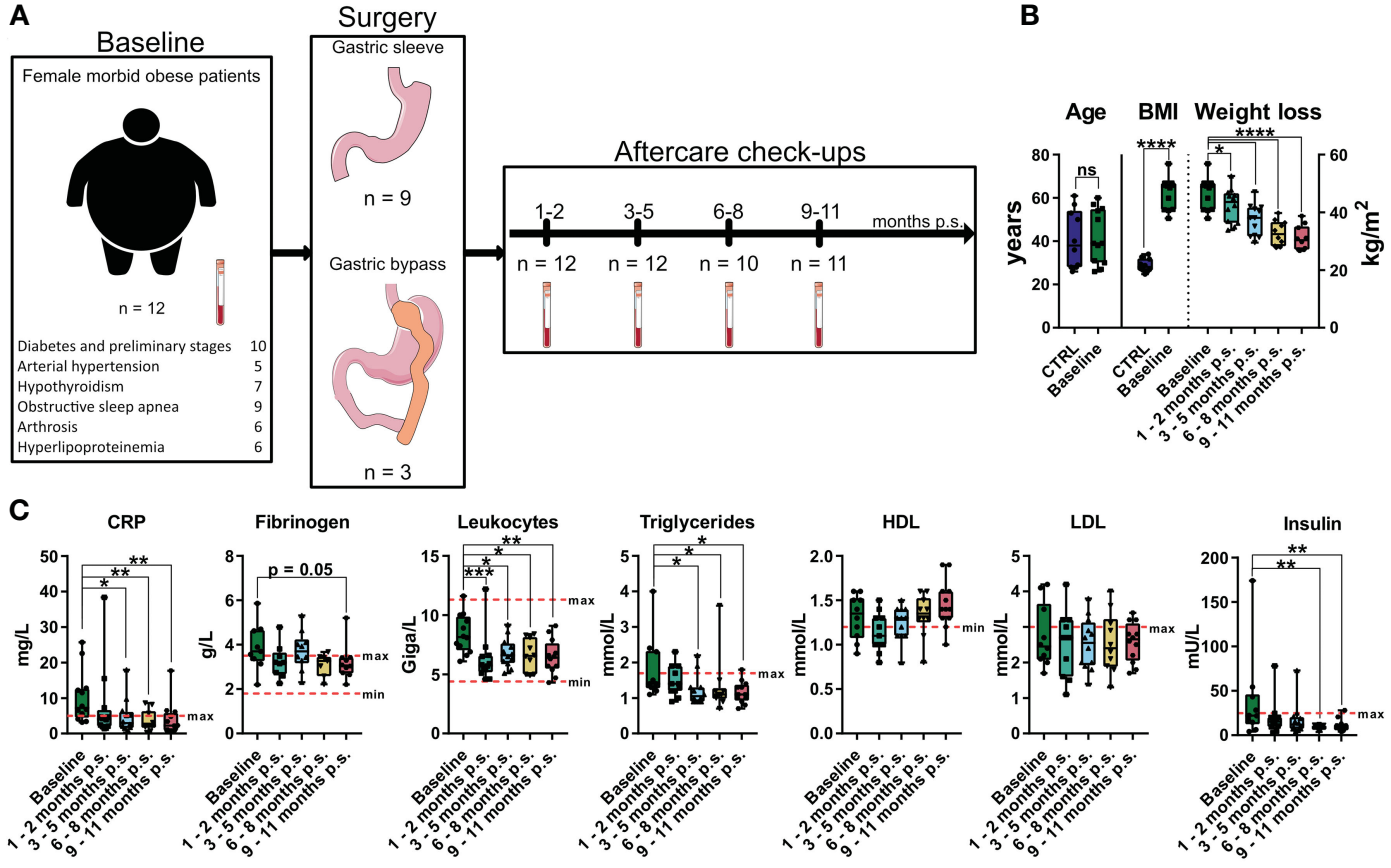
Overview and characterization of the female sub cohort used for high-dimensional mass cytometry analysis. Characterization of weight loss cohort including comorbidities, type of surgery, timepoints of sampling and sample size **(A)** as well as comparison of age and BMI to the control cohort (CTRL) **(B)**. Weight loss illustrated as time-progression of BMI **(B)** as well as time-progression of clinical parameters **(C)** including CRP [mg/L], fibrinogen [g/L], leukocytes [Giga/L], triglycerides [mmol/L], HDL [mmol/L], LDL [mmol/L] and insulin [mU/L] are shown and compared to the baseline values. The clinical minimum and maximum reference values are shown as dotted red lines. Statistical analysis was performed using two-sided unpaired t-test (Age), Welch’s test (BMI), one-way ANOVA with Fisher’s LSD test (weight loss, HDL, LDL) or Kruskal-Wallis test with Dunn’s test (CRP, fibrinogen, leukocytes, triglycerides, insulin) with α = 0.05. *p-value ≤ 0.05, **p-value ≤ 0.01, ***p-value ≤ 0.001, ****p-value ≤ 0.0001. The Figure was partly generated using Servier Medical Art, provided by Servier, licensed under a Creative Commons Attribution 3.0 unported license.

To investigate the influence of bariatric surgery on the health status of patients with morbid obesity a subcohort consisting of 12 female patients with morbid obesity undergoing gastric sleeve or gastric bypass surgery was established (**Figure 2A**). Blood samples were taken prior to surgery (Baseline) and within aftercare checkups 1 - 2 months post-surgery (p.s.), 3 - 5 months p.s., 6 - 8 months p.s., and 9 – 11 months p.s. as illustrated in **Figure 2A**. The previously described gender- and age-matched control cohort (CTRL) was used as a reference for normal BMI (**Figure 2B**), adipocytokine levels, and immune cell levels determined via mass cytometry. For the time progression of weight loss (**Figure 2B**) and clinical blood parameters (**Figure 2C**) values were compared to the baseline.

Compared to baseline, bariatric surgery resulted in significant weight loss (**Figure 2B**) after only 1 - 2 months p.s.. Additionally, compared to baseline, inflammatory blood parameters like CRP significantly decreased within the first 3 – 5 months p.s. and the concentration of circulating leukocytes significantly decreased as soon as 1 – 2 months p.s. (**Figure 2C**), whereas fibrinogen tends to be non-significantly decline 9 – 11 months p.s. Furthermore, metabolic parameters including triglyceride levels as well as the insulin levels significantly declined after surgery, reaching statistical significance 3 – 5 months p.s. (triglycerides) or rather 6 – 8 months p.s. (insulin) compared to baseline. Whereas LDL levels seemed to be less affected by bariatric surgery, HDL concentration slightly elevated 9 – 11 months p.s., without reaching statistical significance compared to baseline (**Figure 2C**).

### 3.3 Bariatric surgery normalizes levels of adipocytokines but unphysiologically reduced ghrelin levels

**Figure 3:**
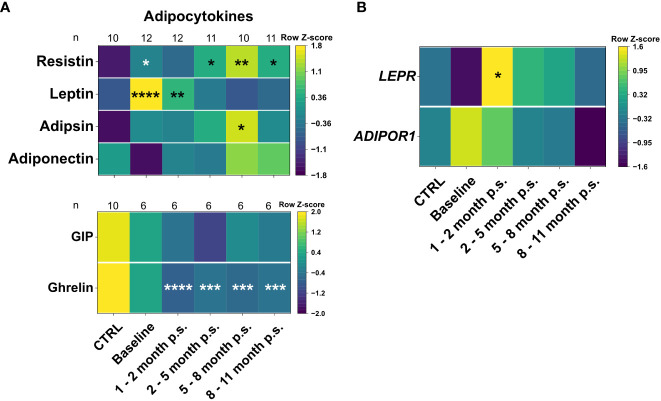
Plasma levels of adipocytokines and gastrointestinal hormones **(A)**, as well as gene expression levels of adipocytokines receptors **(B)** for the CTRL, Baseline, 1 – 2 months p.s., 3 – 5 months p.s., 6 – 8 months p.s. as well as 9 – 11 months p.s. The number of analyzed samples (n) is shown for each group and analysis. Adipocytokines adiponectin, adipsin, leptin and resistin were determined using a bead-based immunoassay and gastrointestinal hormones were determined using ELISAs **(A)**. Gene expression of adipocytokine receptors *ADIPOR1* and *LEPR* was determined with qRT-PCR analysis using *GAPDH* and *RPLP0* as reference genes **(B)**. Data is depicted as time-progression heatmaps whereby each row was normalized by z-score. Statistical analysis was performed compared to the CTRL using a Kruskal-Wallis test with Dunn’s test and α = 0.05. *p-value ≤ 0.05, **p-value ≤ 0.01, ***p-value ≤ 0.001, ****p value ≤ 0.0001.

In order to assess the impact of the bariatric surgery on plasma levels of the adipocytokines and gastrointestinal hormones, bead-based immunoassays, and ELISAs were used to investigate adiponectin, adipsin, leptin and resistin, as well as ghrelin and GIP (**Figure 3A**). Moreover, the gene expression levels of the respective adipocytokine receptors *ADIPOR1* and *LEPR* were analyzed in whole blood samples (**Figure 3B**). A healthy, lean (BMI ≤ 25 kg/m^2^), gender- and age-matched control cohort (CTRL, n = 10) (**Figure 2B**) was used to determine reference levels. Regarding the adipocytokine levels, adiponectin tend to be non-significantly lower at baseline compared to the CTRL but normalized after 1 – 2 months p.s.. However, adipsin did not show any alteration at baseline compared to the CTRL. Moreover, leptin was significantly elevated at baseline compared to the CTRL and continuously declined to reach CTRL levels at 2 – 5 months p.s.. Apart from that, resistin was slightly increased at baseline compared to the CTRL but did normalize within 9 – 11 months p.s. Interestingly, gene expression of the receptors *ADIPOR1* and *LEPR* (**Figure 3B**) seemed to be regulated in an opposite manner regarding the respective protein levels. The gene expression of *ADIPOR1* seemed to be slightly, but non-significantly, upregulated in the peripheral blood immune cells of patients with morbid obesity compared to the CTRL but reached CTRL levels at 3 – 5 months p.s.. Similarly, *LEPR* expression level tended to be slightly, but non-significantly, decreased in the peripheral blood immune cells of patients with morbid obesity compared the CTRL but showed significantly increased levels 1 – 2 months p.s., reaching CTRL levels 9 – 11 months p.s. Ghrelin levels were already slightly, non-significantly decreased at baseline compared to the CTRL and further decreased after surgery not showing any sign of rising to CTRL levels within the observed period. Apart from that, GIP levels seemed to be slightly, but non-significantly, decreased in patients with morbid obesity compared to the CTRL and were not affected by bariatric surgery.

### 3.4 Mass cytometry revealed alterations in main innate and adaptive immune cell compartments of patients with morbid obesity

**Figure 4:**
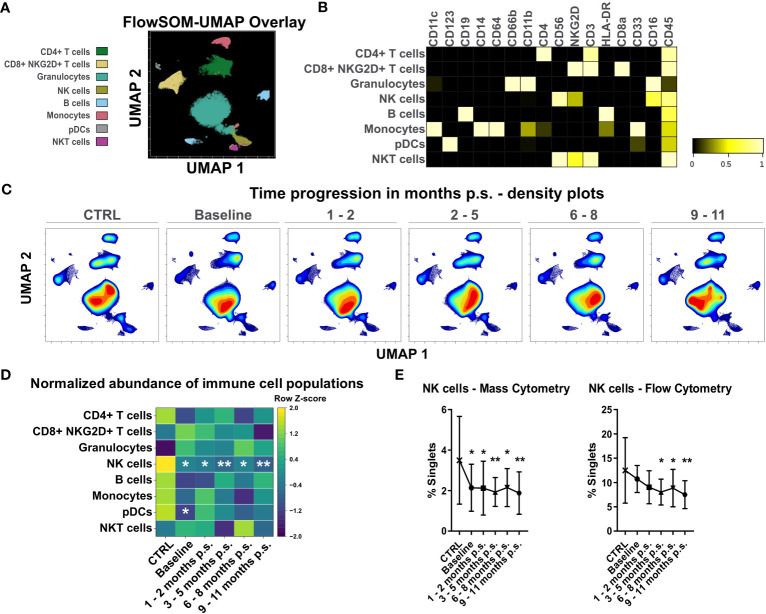
Analysis of whole blood samples using high-dimensional mass cytometry. FlowSOM-UMAP Overlay with 20,000 cells per sample using the markers CD11c, CD123, CD19, CD14, CD64, CD66b, CD11b, CD4, CD56, NKG2D, CD3, HLA-DR, CD8a, CD33, CD16 and CD45 was performed in Cytobank and enabled identification of main peripheral blood immune cell populations **(A)**. Manually annotated cell populations were confirmed by evaluating surface marker expression **(B)**. Processed files of the same group were concatenated and illustrated as UMAP density plots over time for CTRL, baseline, 1 - 2 months p.s., 3 - 5 months p.s., 6 - 8 months p.s. and 9 - 11 months p.s. **(C)**. Abundances of identified immune cell populations are depicted as time-progression heatmaps whereby each row was normalized by z-score **(D)**. Cross verification was performed by comparing time-progression of NK cells analyzed with mass cytometry and flow cytometry (mean ± SD) **(E)**. Statistical analysis was performed compared to the CTRL using one-way ANOVA with Fisher’s LSD test with α = 0.05. *p-value ≤ 0.05, **p-value ≤ 0.01.

High-dimensional mass cytometry was used to analyze peripheral blood immune cell populations in 12 female patients with morbid obesity undergoing bariatric surgery (**Figure 2A**). A healthy, lean (BMI ≤ 25 kg/m^2^), gender- and age-matched control cohort (CTRL, n = 10) (**Figure 2B**) was used to determine reference levels. Major immune cell populations were detected using dimensionality reduction *via* UMAP and clustering with FlowSOM algorithm (**Figure 4A**). Clusters were manually annotated according to the expression patterns of surface markers CD45, CD66b, CD3, CD4, CD8a, NKG2D, CD19, CD33, CD56, HLA-DR, CD16, CD14, CD64, CD11b, CD11c, CD123 (**Figure 4B**). Illustration of UMAP density plots revealed shifts in the major immune cell populations of patients with morbid obesity compared to the CTRL cohort conspicuously visible in CD4+ T cells, granulocytes and monocytes (**Figure 4C**). Additionally, abundances of the detected immune cell populations normalized to the respective mean of the CTRL population were determined (**Figure 4D**). Using mass cytometry, NK cells (Lin-, CD16^+^, CD56^+^, NKG2D^+^) were shown to be significantly decreased in the condition of morbid obesity compared to the CTRL population. Moreover, bariatric surgery led to a further decline of the NK cell population which did not recover within 9 – 11 months p.s. (**Figure 4E**). Cross verification was performed using conventional flow cytometry to confirm the results obtained from mass cytometry (**Figure 4E**).

Due to the constant decrease of NK cell levels after surgery and the visible shifts in CD4+ T cells and monocytes, that were also detectable by flow cytometry, these immune cell populations were further investigated using the Spectre R package.

### 3.5 Morbid obesity led to a shift from naïve to memory CD4+ T cells, which was not rescued by bariatric surgery within 9 – 11 months p.s.

**Figure 5:**
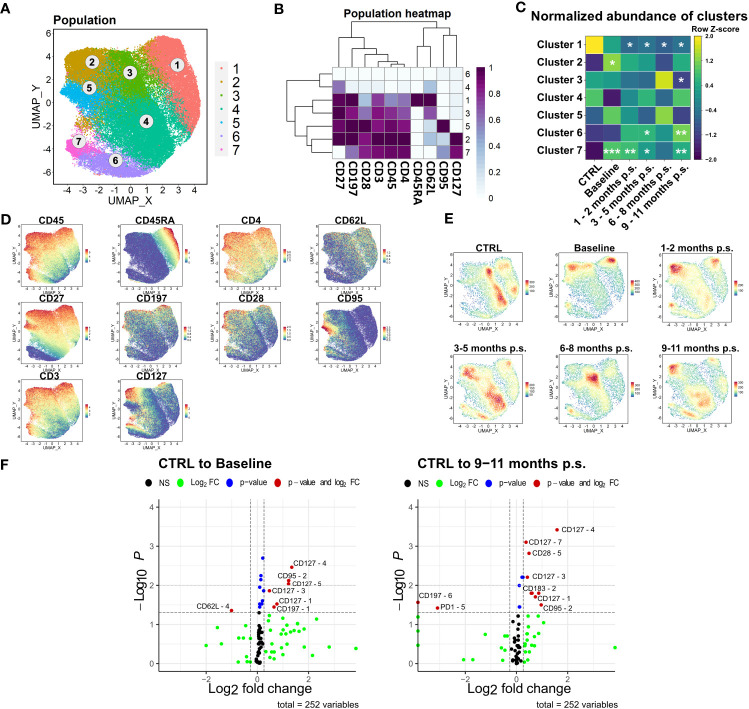
Detailed analysis of CD4+ T cell subsets. FlowSOM-UMAP Overlay with 10,000 cells per group using the markers CD27, CD197, CD28, CD3, CD45, CD4, CD45RA, CD62L, CD95 and CD127 was performed following the Spectre R script **(A)**. Expression heatmap **(B)** as well as expression patterns of cluster markers **(D)** enabled identification of 7 distinct clusters. Abundances of identified immune cell populations are depicted as time-progression heatmaps whereby each row was normalized by z-score **(C)**. Statistical analysis was performed compared to the CTRL using Kruskal-Wallis test with Dunn’s test and α = 0.05. *p-value ≤ 0.05, **p-value ≤ 0.01, ***p-value ≤ 0.001 **(C)**. Density plots showed development and shifts within clusters for CTRL, baseline, 1 - 2 months p.s., 3 – 5 months p.s., 6 – 8 months p.s. as well as 9 – 11 months p.s **(E)**. Volcano plots (Log_2_ fold change cut off = 0.26, p-value cut off = 0.05, p-value calculation = Wilcoxon test) indicated differentially expressed surface markers on identified clusters at baseline and 9 – 11 months p.s. compared to the CTRL **(F)**.

The shift observed in the CD4+ T cells was analyzed at a higher resolution using the Spectre R package (30). Prior to the analysis, manually gated CD4+ T cells were compared to the CD4+ T cell population identified with unsupervised clustering using FlowSOM (**Figure 4A**) to verify the defined populations (**Supplementary Figure 3**). Linear regression analysis (Slope = 0.9584, R^2^ = 0.9915) showed a high conformity of manual and automated gating. Accordingly, manually gated CD4+ T cells were selected for further analysis and clustered using FlowSOM followed by visualization with UMAP (**Figure 5A**) using the markers CD45, CD3, CD4, CD45RA, CD197, CD27, CD127, CD95, CD62L and CD28. Seven clusters were defined according to different surface marker expression (**Figures 5B–D**). Cluster 1 identified CD45RA^+^, CD62L^+^, CD27^+^ and CD197^+^ naïve T cells (34). Furthermore, cluster 2, 3, 4 and 5 identified CD45RA^-^, CD62L^+^, CD27^+^ central memory T cells, whereas cluster 6 and 7 identified CD45RA^-^, CD62L^-^ and CD27^-^ effector memory T cells (35, 36). The time-progression of the density plots (**Figure 5E**) revealed shifts in the mentioned CD4+ T cell subpopulations when comparing CTRL and baseline. Naïve T cell levels (Cluster 1) tend to be slightly, but non-significantly, decreased in patients with morbid obesity compared to the CTRL population and further declined after surgery reaching significantly decreased levels 1 – 2 months p.s. (**Figure 5C**). In contrast, CD127^high^ central memory (Cluster 2) and CD127^high^ effector memory (Cluster 7) T cell subsets were significantly expanded in patients with morbid obesity. Interestingly, 1 – 2 months p.s. both subsets already decreased, reaching a minimum at 6 – 8 months p.s., also detectable in the corresponding density plots. Nevertheless, 9 – 11 months p.s., a disturbance of the CD4+ T cell compartment was still detectable characterized by decreased naïve T cells and increased effector memory subsets (Cluster 6 and 7).

According to the resulting volcano plots (**Figure 5F**), an elevated expression of CD127 could be observed in several distinct clusters in patients with morbid obesity, even 9 – 11 months p.s., hinting towards a possible obesity-associated dysfunction within the regulation of this receptor.

### 3.6 NK cells of patients with morbid obesity showed increased expression of activation markers positively affected by bariatric surgery

**Figure 6:**
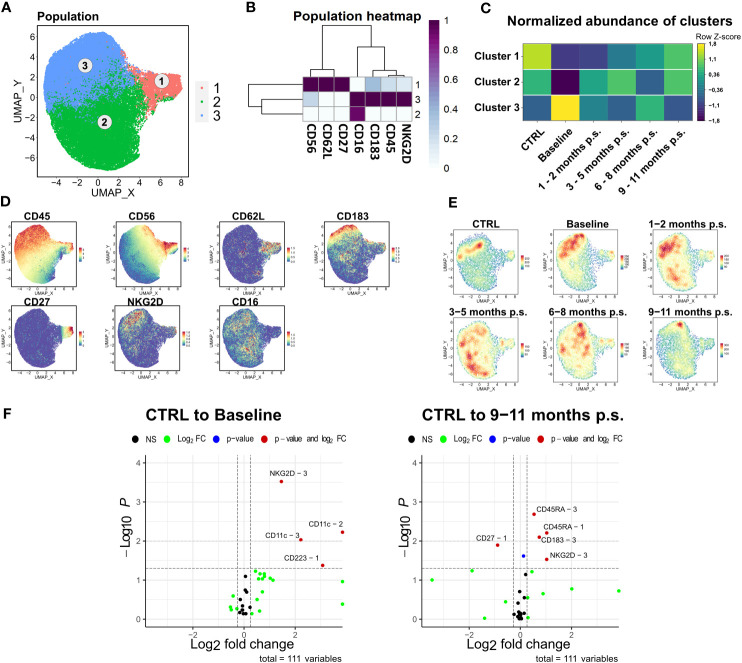
Detailed analysis of NK cell subsets. FlowSOM-UMAP Overlay with 10,000 cells per group using the markers CD56, CD62L, CD27, CD16, CD183, CD45 and NKG2D was performed following the Spectre R script **(A)**. Expression heatmap **(B)** as well as expression patterns of cluster markers **(D)** enabled identification of three distinct clusters. Abundances of identified immune cell populations are depicted as time-progression heatmaps whereby each row was normalized by z-score. Statistical analysis was performed compared to the CTRL using Kruskal-Wallis test with Dunn’s test and α = 0.05 **(C)**. Density plots showed development and shifts within clusters for CTRL, baseline, 1 – 2 months p.s., 3 – 5 months p.s., 6 – 8 months p.s. as well as 9 – 11 months p.s. **(E)**. Volcano plots (Log_2_ fold change cut off = 0.26, p-value cut off = 0.05, p-value calculation = Wilcoxon test) indicated differentially expressed surface markers on identified clusters at Baseline and 9 – 11 months p.s. compared to the CTRL **(F)**.

As the ratio of NK cells significantly decreased after bariatric surgery, the NK cell population was further analyzed. Comparable to the workflow described for CD4+ T cell, manually gated NK cells were compared to the NK cells identified via unsupervised clustering using FlowSOM (**Supplementary Figure 3**). Manually gated NK cells were clustered using FlowSOM followed by visualization with UMAP (**Figure 6A**) using the markers CD45, CD56, CD62L, CD183, CD27, NKG2D, and CD16. Three distinct clusters were defined according to their surface marker expression (**Figures 6B–D**). Cluster 1 defined the CD56^high^, CD16^low^ cytokine-producing NK cell subset, whereas cluster 2 and 3 defined the CD56^dim^, CD16^+^ cytolytic NK cell subset (37, 38). Apart from that, the CD56^high^ NK cell subset was described to express CD62L and CD27 (39). The cytotoxic NK cell subset could be further distinguished by the expression of NKG2D (cluster 3) (40), which is known to be an activating cell surface receptor (41). Regarding the density plot (**Figure 6E**), a considerable disturbance could not be observed in the NK cells department. The normalized abundances of the mentioned clusters (**Figure 6C**) revealed a non-significant shift towards CD56^dim^, CD16^+^, NKG2D^+^ in patients with morbid obesity, which normalized rapidly after bariatric surgery. Additionally, the expression levels of NKG2D, CD11c, and CD223, also known as lymphocyte activation gene-3 (LAG-3), were significantly increased in the CD56^dim^, CD16^+^, NKG2D^+^ subset (**Figure 6F**) from patients with morbid obesity compared to the CTRL population. Within 9 – 11 months p.s., the expression of CD223 normalized in the CD56^dim^, CD16^+^ NKG2D^+^, whereas the expression of NKG2D was still slightly elevated.

### 3.7 Patients with morbid obesity show disturbance in monocyte compartment, which is partially restored within 9 – 11 months p.s.

**Figure 7:**
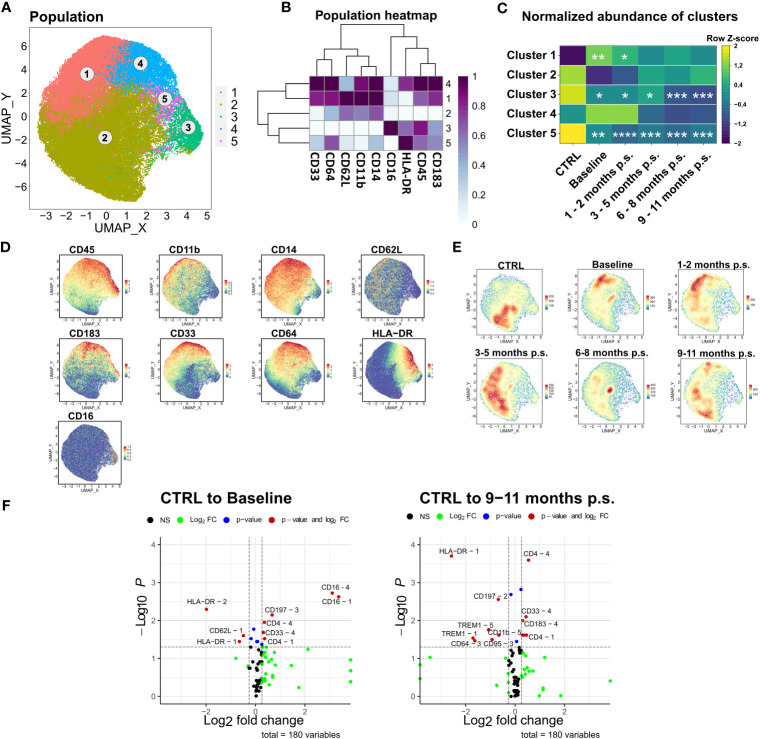
Detailed analysis of monocyte subsets. FlowSOM-UMAP Overlay with 10,000 cells per group using the markers CD33, CD64, CD62L, CD11b, CD14, CD16, HLA-DR, CD45 and CD183 was performed following the Spectre R script **(A)**. Expression heatmap **(B)** as well as expression patterns of cluster markers **(D)** enabled identification of 5 distinct clusters. Abundances of identified immune cell populations are depicted as time-progression heatmaps whereby each row was normalized by z-score **(C)**. Statistical analysis was performed compared to the CTRL using one-way ANOVA with Fisher’s LSD test and α = 0.05. *p-value ≤ 0.05, **p-value ≤ 0.01, ***p-value ≤ 0.001, ****p-value ≤ 0.0001 **(C)**. Density plots showed development and shifts within clusters for CTRL, baseline, 1 – 2 months p.s., 3 – 5 months p.s., 6 – 8 months p.s. as well as 9 – 11 months p.s. **(E)**. Volcano plots (Log2 fold change cut off = 0.26, p-value cut off = 0.05, p value calculation = Wilcoxon test) indicated differentially expressed surface markers on identified clusters at Baseline and 9 – 11 months p.s. compared to the CTRL **(F)**.

The last subset that was investigated more specifically was the monocyte compartment. As previously described, manually gated monocytes were compared to the monocyte population determined via unsupervised clustering (**Supplementary Figure 3**). The manually gated monocytes were clustered into five distinct clusters based on surface marker expression of CD33, CD64, CD62L, CD11b, CD14, CD16, HLA-DR, CD45, and CD183 (**Figures 7A–D**). Cluster 1 and 2 defined monocytes with lower HLA-DR expression but higher expression of CD62L. CD62L is a recruitment marker highly expressed on the classical monocyte subsets (42). Compared to cluster 2, cluster 1 showed higher CD183, CD11b, and CD33 expression and was significantly increased in patients with morbid obesity compared to the CTRL. Moreover, CD183, also known as CXCR3, is a chemokine receptor involved in the migration of monocytes into inflamed tissue (42, 43). Cluster 1, representing a CD183^+^ CD62L^+^ monocyte subset, was significantly enlarged in patients with morbid obesity but slowly diminished after bariatric surgery (**Figure 7C**). Besides that, cluster 3 (CD16^+^) and cluster 5 representing low expression of CD14 and CD62L, as well as high expression of HLA-DR were significantly decreased in patients with morbid obesity most likely due to the observed shift towards cluster 1 (**Figure 7E**). However, these cells did not return to CTRL levels after surgery. Moreover, a significantly decreased expression of the surface marker HLA-DR was observed on cluster 1 and 2 (**Figure 7F**). Importantly, the significant decrease in HLA-DR expression on the monocytes of patients with morbid obesity was also detectable in the flow cytometry data (data not shown). After 9 – 11 months p.s., cluster 1 kept the low HLA-DR expression level. Nevertheless, a decreased expression of TREM1, an activating receptor of monocytes that is involved in mediating inflammation (44), was observed in cluster 1 and 5 at 9 – 11 months p.s.

## 4 Discussion

In this study, the influence of bariatric surgery on the peripheral blood immune cells of female patients with morbid obesity was investigated within the first year post-surgery using high-dimensional mass cytometry. Here, the observations were related to a healthy, lean, gender- and age-matched control group.

The patients showed typical signs of obesity-associated low-grade chronic inflammation characterized by elevated CRP, fibrinogen, and leptin levels and decreased levels of adiponectin (45). After surgery a rapid adaption of several inflammation-associated and metabolic blood parameters including CRP, fibrinogen, leukocyte count, triglycerides, HDL, LDL, and insulin could be observed as soon as 1 – 2 months p.s. Furthermore, the adaptions in adipocytokine levels also hint towards a fast relief of the chronic inflammation as a result of bariatric surgery as leptin and adiponectin both show immunostimulatory functions. Leptin was described as a pro-inflammatory factor inhibiting NK cells and inducing the proliferation and activation of monocytes (46). Adiponectin was shown to have anti-inflammatory properties (47) and low levels were associated with chronic inflammation (48). Moreover, not only the protein levels adapted after surgery but also the gene expression levels of the respective receptors *ADIPOR1* and *LEPR*. Interestingly, in a short-term reaction up to 1 – 2 months p.s., the gene expression seemed to change in a reciprocal manner regarding the circulating protein levels. Whether this might be due to up- or down-regulation of the receptors as a reaction to altered protein levels as it was shown for insulin (49) or due to an altered cell composition accumulating or diminishing cell populations expressing these receptors remains to be elucidated. Taken together, bariatric surgery was shown to be highly effective in resolving the obesity-associated low-grade chronic inflammation and rapidly normalized lipid and adipocytokine levels as soon as 1 – 2 months p.s., indicating weight loss-independent mechanisms. Moreover, peripheral blood immune cells were shown to express adipocytokine receptors highlighting a possible way of interaction contributing to the dysregulation of immune cells in obesity but also to the observed adaptions after surgery.

Apart from adipocytokines, circulating levels of the gastrointestinal hormone ghrelin have been investigated. Ghrelin levels were decreased in patients with morbid obesity and further decreased after surgery, which might be beneficial for weight loss after surgery as ghrelin has stimulatory effects on food intake and fat deposition (50). Nevertheless, ghrelin was shown to possess strong anti-inflammatory and antioxidative properties (51, 52), as well as promoting lymphocyte development in thymus (53). Thereby, ghrelin was shown to recover function and thymopoiesis in an aged thymus (54, 55). Accordingly, on the one hand, unphysiological low levels of ghrelin after surgery might contribute to successful long-term weight loss after surgery but on the other hand could contribute to a delayed regeneration of the immune system.

Although the patients already lost a significant amount of weight 1 – 2 months p.s., it is still unclear whether these rapid metabolic improvements are rather a consequence of the surgical procedure itself than a direct effect of weight loss (56, 57). However, these rapid adaptions are considered as one of the major advantages of bariatric surgery.

Taking peripheral blood immune cell compartments into account, the patients with morbid obesity demonstrated a considerable increase of a Lin^-^, HLA-DR^-^, CD14^+^ sub-population, which phenotypically represents monocytic Myeloid derived suppressor cells (mo-MDSCs) (58, 59). Obesity-derived mo-MDSCs were described to be linked to an increased cancer risk that occurs in obese patients as they promote tumor progression and trigger apoptosis in tumor-infiltrating CD8+ T cells (60, 61). Furthermore, recent data suggested that in a state of obesity, long term exposure to metabolic factors like polyunsaturated fatty acids favors the differentiation of MDSCs from bone marrow precursors and lead to a metabolic reprogramming restricting their responsiveness (62, 63). Interestingly, we also observed a significant increase in a monocytic cluster containing HLA-DR^-^, CD14^+^, CD11b^+^ cells, that additionally express CXCR3 and CD62L. Both receptors were shown to be involved in the recruitment of monocytes to inflamed or tumor-bearing tissue (64), and therefore might be involved in monocytes accumulation in adipose and tumor tissue observed in obesity (65, 66). Indeed, the number of CD11b^+^ cells was significantly decreased in the adipose tissue of CXCR3^−/−^-high-fat diet (HFD) mice compared to wild type-HFD mice (67). Similarly, an increase in the percentages of monocytes was observed in female obese individuals compared to controls due to enhanced intrinsic migratory capacity of peripheral monocytes (68). Interestingly, our results further confirmed these findings, as the percentage of the mo-MDSCs cells expressing CXCR3 and CD62L was rapidly diminished after surgery, supporting the correlation between peripheral immune cell dysfunction and obesity. Hence, this adaption might contribute to the reduction in risk of developing obesity-associated cancers that has been observed in obese patients after surgery (69). Apart from the observed shifts in cellular composition, we found a decreased HLA-DR expression of the monocytic subset, which was not fully reversed within 9 – 11 months p.s. Reduced HLA-DR expression on monocytes reflects a state of impaired immunity and immunosuppression (70) and was shown to be closely related to cholesterol and triglyceride levels in diabetic patients (71). Further, obese patients showed an impaired recovery of monocytic HLA-DR after surgery, which was associated with a higher risk of sepsis (72). Summarized, patients with morbid obesity showed a disturbed monocytic compartment characterized by high levels of mo-MDSCs and decreased monocytic HLA-DR expression indicating a state of immunosuppression and impaired immunity. Although bariatric surgery decreased levels of mo-MDSCs, HLA-DR expression was not recovered within 9 – 11 months p.s. possibly influencing the immune response of patients within this period.

Apart from alterations in the monocyte compartment, differences in the CD4+ T cell population characterized by decreased naïve T cells and increased memory T cells have been described in obesity (2). It was shown that the thymic function was highly impaired in obese mice leading to decreased naïve T cells and expanded memory T cells. Further, these results were transferred to humans indicating that obesity accelerates thymic aging reflected by the inability of the thymus to replenish the naïve T cell pool and therefore increasing the risk of infections (13). One important characteristic of an aged thymus is the expansion of adipocytes and its transformation into adipose tissue contributing to the loss of thymic functionality and impairing T cell development (73). Likely, obesity accelerates this process by elevating infiltrating adipocytes and increasing the accummulation of perithymic adipose tissue as it was shown in high-fat diet induced obese mice (13). Indeed, it was observed that in a young human population thymic fat infiltration was associated with the BMI (74). Thymic adipocytes might disturb the thymic secretome releasing thymic suppressive factors like leukemia inhibitory factor and simultaneously contributing to the reduction of critical thymic growth factors like stem cell factor, fibroblast growth factors 7 and 10 as a consequence of a thymic fibroblast-to-adipocyte transition (75). Interestingly, the naïve T cells did not recover within the first year post-surgery. Ghrelin might be one factor involved in the observed disturbance of the T cell compartment. It was shown that the ghrelin receptor was highly expressed on developing murine thymocytes but the thymic expression of ghrelin ligand and receptor decreased with aging. Interestingly, ghrelin infusion recovered the age-related thymic involution increasing lymphoid progenitors and reduced splenic and thymic macrophage numbers (54, 55, 76). Furthermore, it was shown that the genetic ablation of ghrelin ligand and ghrelin receptor in the thymus of mice led to epithelial-mesenchymal transition as well as thymic adipogenesis and was also associated to decreased naïve T cells (77). According to this, the decreased circulating ghrelin levels observed in patients with morbid obesity and the further decline after bariatric surgery could be involved in thymic dysfunction in these patients and contribute to the observed disturbance in the T cell compartment. However, data of 6 patients at a later time point (15 – 19 months p.s.) indicated the recovery of the naïve T cell pool eventually hinting towards a long-term regeneration of the thymus (**Supplementary Figure 5**). Here we observed a decrease of naïve T cells together with an increase of two distinct clusters of central memory and effector memory T cells, especially characterized by a high expression of CD127. CD127 or IL-7Rα plays a major role in T-cell survival, maturation, as well as homeostasis (78) and was described to be a marker for long-living memory T cells (79). Therefore, CD127 might also be highly involved in the observed disruption of T cell homeostasis in obesity. Possible mechanisms that have been described to be responsible for the expansion of the memory T cell pool include the dysregulation of IL-7Rα, an increased turnover rate of naïve T cells favoring their conversion into memory T cells as well as the increased availability of IL-7 or IL-15 as a consequence of a diminished naïve T cell population (80). Interestingly, memory T cells expressing high levels of IL-7Rα were shown to be drivers of colitis in mice and could be maintained and expanded with IL-7 (81). Moreover, blockage of IL-7Rα was shown to control inflammation in primates via neutralization of antigen-specific memory T cell subsets (82). In this context, it is important to mention that IL-7 levels were shown to be increased in patients with morbid obesity (83). Since IL-7R is also expressed on early B cells and its expression and function is critical for proper lymphocyte development (84), blocking this receptor may affect other normal immune cells. For instance, previous report showed that *Il7r* deficient mice exhibited depletion in both T and B lymphocytes (85). Another study showed that IL-7Rα mutations in humans result in severe combined immunodeficiency (SCID), which is characterized by the lack of T cells and normal numbers but dysfunctional B cells (86).

Accordingly, targeting IL-7Rα using specific antibodies may also affect B cells and result in immunodeficiency in obese patients. However, a recent study showed that treating healthy individuals with anti-human IL-7R antibody was well tolerated and did not lead to apparent alterations in immune cell compartments and inflammatory cytokine profiles (87). Thus, blocking IL7-R signaling might provide a key therapeutic approach to impact survival of IL-7R expressing memory T cells, improving T cell homeostasis, and controlling inflammation in a state of morbid obesity. Although this effect only appears after more than one year p.s., bariatric surgery might lead to a more long-lasting improvement of the T cell compartment by reversing and recovering thymic function.

Next, we showed that NK cells were decreased in patients with morbid obesity and further decreased after bariatric surgery. However, we could not find significant shifts in abundances within NK cell subsets. Several studies described decreased levels of NK cells in patients with morbid obesity coming along with an increased activation status due to dysregulation of activation and inhibitory molecules as well as a lack of function including restricted antitumor response (14, 88, 89). However, there are only a few studies investigating the effect of bariatric surgery on the NK cell compartment. It was shown that bariatric surgery improved NK cell activity and increased NK cell cytokine production within 6 months p.s (22). Nevertheless, data regarding NK cell abundance is contradictory most likely due to different time-points investigated (89).

Within this study, few limitations should be mentioned. First, the study only includes female patients with morbid obesity, as 75% of the patients that undergo bariatric surgery in our department are women. Nevertheless, this deprives the opportunity to consider sex-specific differences regarding levels of sex-hormones or the immune response (90) and their influence on the state of obesity and the outcome of bariatric surgery. However, the strict diet patients received prior to surgery might influence the immune system as well as the obesity homeostasis. Therefore, taking and analyzing samples prior to the start of the diet might increase the power of this kind of studies. Furthermore, it might be worth to increase the observed time-period after surgery. Although the patients lost a significant amount of weight within the observed time-period, none of them reached a BMI of ≤ 25 kg/m^2^. Although most of the parameters including CRP, fibrinogen, adipocytokine levels and some immune cell populations seem to rapidly adapt after surgery, alterations are still detectable 9 – 11 months p.s., especially with regard to the expression of activation markers and NK cell levels. Here, additional time points at 18 or 24 months p.s. might help to clarify or exclude irreversible alterations as a result of long-standing morbid obesity. Due to the relatively small subcohort that was investigated with mass cytometry, a comparison of the surgical procedures on the outcome of bariatric surgery was not possible. Within recent years, laparoscopic sleeve gastrectomy became the predominant bariatric surgical procedure performed (91), most likely because it is considered as technically less demanding compared to gastric bypass (92). Although the likelihood of complications occurring after bariatric surgery is generally considered low, the risk of severe post-surgical complications was lower after sleeve gastrectomy compared to gastric bypass (93). Regarding the effectiveness, it was shown that sleeve gastrectomy and gastric bypass lead to highly comparable improvement with regard to weight loss, remission of DM2, and adaptions in gastrointestinal and pancreatic peptide hormones (94–96). Specifically, gastric sleeve and gastric bypass comparably decreased leptin and ghrelin levels and increased post-prandial GLP-1, PYY, and general bile acid levels contributing to improved insulin sensitivity. Although both procedures were shown to increase HDL levels, gastric bypass seemed to be more effective in reducing LDL and total cholesterol levels (97, 98). Regarding the question of how bariatric surgery impacts immune cell composition and activation, studies that consider different procedures separately are still lacking. A recent study showed that gastric bypass was shown to temporarily reverse obesity-associated accelerated CD4^+^ T cell aging (99). However, patients undergoing gastric sleeve surgery were not included in this study. Apart from that, bariatric surgery was shown to normalize B cell but not T cell composition compared to a lean control cohort. However, cytokine-producing capacity of CD4^+^ T cells was restored after surgery. Nevertheless, a separate evaluation of patients undergoing gastric sleeve and gastric bypass was not performed (2). Consequently, there is a huge demand for studies that investigate the influence of bariatric surgery on the immune system, considering the type of surgery.

In conclusion, this study shows systemic effects of morbid obesity characterized by persistence of a low-grade chronic inflammation and a dysregulation of lipids, adipocytokines and gastrointestinal hormone ghrelin as well as disturbed peripheral blood immune cells indicated by increased levels of mo-MDSCs, decreased NK cells, and decreased levels of CD4^+^ naïve T cells. Nonetheless, the power of bariatric surgery, to not only reduce weight but also effectively improve metabolic and immunological disorders is also demonstrated here. Bariatric surgery rapidly released the low-grade chronic inflammation, normalized adipocytokine levels and decreased the levels of mo-MDSCs. Nevertheless, ghrelin levels, monocytic HLA-DR expression, CD4^+^ naïve T cell, and NK cell levels did not normalize within the observed period of 9 – 12 months p.s. However, data indicated an increase of CD4^+^ naïve T cells 15 – 19 months p.s., indicating a possible regeneration of the immune system at later time points.

## Data availability statement

The raw data supporting the conclusions of this article will be made available by the authors, without undue reservation.

## Ethics statement

The studies involving human participants were reviewed and approved by ethics committee at Ulm University. The patients/participants provided their written informed consent to participate in this study.

## Author contributions

UK, LE and MK contributed the study design and supervision. LE performed the surgery, sampling as well as patient monitoring and follow-up care. LM performed sample processing, RNA isolation and qRT-PCR analysis. AR performed ELISAs. FG and AG performed bead-based immunoassays, flow cytometry and mass cytometry analysis. HA performed flow cytometry analysis. AG performed data analysis and statistical analysis. AG, UK and HA wrote the manuscript. All authors contributed to the article and approved the submitted version.
