# Supplementary material for: Influence of bariatric surgery on the peripheral blood immune system of female patients with morbid obesity revealed by high-dimensional mass cytometry
Source: Front Immunol. 2023 May 11;14:1131893. doi: 10.3389/fimmu.2023.1131893 (PMC10230950; doi:10.3389/fimmu.2023.1131893)
Supplement: Supplementary file 1 [file DataSheet_1.pdf]

Supplementary Material

Influence of bariatric surgery on the peripheral blood immune system of female morbid obese patients revealed by high-dimensional mass cytometry

Adrian Gihring[1], Fabian Gärtner[1], Laura Mayer[1], Aileen Roth[1], Hend Abdelrasoul[1], Marko Kornmann[1], Leonard Elad[1†], and Uwe Knippschild[1*†]

*** Correspondence:** Uwe Knippschild: uwe.knippschild@uniklinik-ulm.de

1 Supplementary Data

Suppl. Table 1: Characteristics of female CTRL cohort (n = 10) including age, body mass index (BMI) and blood parameters. The values determined in serum samples in the central institution of clinical chemistry at Ulm University Hospital.

Female cohort – CTRL<br>n = 10		
**Characteristics**	**Median [Min. – Max.]**	
Age	38 [26 – 61]	
BMI [kg/m^2]	21.1 [18.6 – 25.0]	
**Blood parameter**		
**Parameter [Unit]**	**Median [Q1 – Q3]**	**n**
CRP [mg/L]	0.61 [0.60 – 1.70]	10
Triglycerides [mmol/L]	0.77 [0.60 – 1.28]	10
Cholesterol [mmol/L]	4.80 [4.36 – 5.99]	10
HDL [mmol/L]	1.94 [1.78 – 2.25]	10
LDL [mmol/L]	2.57 [2.32 – 3.99]	10
AST [U/L]	19.60 [17.70 – 26.63]	10
ALT [U/L]	17.60 [12.48 – 20.30]	10
Uric acid [µmol/L]	264.50 [204.30 – 312.00]	10
AP [U/L]	66.85 [51.93 – 81.65]	10
CK [U/L]	94.25 [62.48 – 161.50]	10
LDH [U/L]	164.00 [142.30 – 190.50]	10
Lipase [U/L]	40.00 [30.25 – 60.45]	10
Insulin [mU/L]	11.44 [4.91 – 48.75]	10

Suppl. Table 2: Antibodies used for surface staining of mass cytometry samples.

Isotope	Antigen	Clone	Company	Product#	Dilution
89Y	CD45	HI30	Fluidigm	3089003B	1:100
141Pr	CD196	G034E3	Fluidigm	3141003A	1:500
142Nd	CD66b	REA306	Miltenyi	130-108-019	1:5000
143Nd	CD45RA	HI100	Fluidigm	3143006B	1:500
144Nd	CD11b	IRCF44	Fluidigm	3144001B	1:500
145Nd	CD4	RPA-T4	Fluidigm	3145001B	1:300
146Nd	IgD	IA6-2	Fluidigm	3146005B	1:500
147Sm	CD11c	Bu15	Fluidigm	3147008B	1:300
148Nd	CD14	RMO52	Fluidigm	3148010B	1:500
149Sm	CD56	NCAM16.2	Fluidigm	3149021B	1:500
150Nd	CD223	11C3C65	Fluidigm	3150030B	1:500
151Eu	CD123	6H6	Fluidigm	3151001B	1:500
152Sm	TCRgd	11F2	Fluidigm	3152008B	1:500
153Eu	CD62L	DREG56	Fluidigm	3153004B	1:500
154Sm	Tim-3	F38-2E2	Fluidigm	3154010B	1:500
155Gd	CD279/PD1	EH12.2H7	Fluidigm	3155009B	1:500
156Gd	CD183	G025H7	Fluidigm	3156004B	1:500
158Gd	CD27	L128	Fluidigm	3158010B	1:500
159Tb	CD197	G043H7	Fluidigm	3159003A	1:500
160Gd	CD28	CD28.2	Fluidigm	3160003B	1:500
161Dy	CD80	2D10.4	Fluidigm	3161023B	1:500
162Dy	CD8a	RPA-T8	Fluidigm	3162015B	1:300
163Dy	CD33	WM53	Fluidigm	3163023B	1:500
164Dy	CD95	DX2	Fluidigm	3164008B	1:500
165Ho	CD19	HIB19	Fluidigm	3165025B	1:500
166Er	CD314/NKG2D	ON72	Fluidigm	3166016B	1:500
167Er	CD64	REA978	Miltenyi	130-124-325	1:500
168Er	CD154/CD40L	24-31	Fluidigm	3168006B	1:500
169Tm	CD25	2A3	Fluidigm	3169003B	1:500
170Er	CD3	UCHT1	Fluidigm	3170001B	1:500
171Yb	CD20	2H7	Fluidigm	3171012B	1:500
172Yb	CD354/TREM1	TREM-26	Fluidigm	3172022B	1:500
174Yb	HLA-DR	L243	Fluidigm	3174001B	1:500
175Lu	CD274/PDL1	29E.2A3	Fluidigm	3175017B	1:500
176Yb	CD127	A019D5	Fluidigm	3176004B	1:500
209Bi	CD16	3G8	Fluidigm	3209002B	1:500

Suppl. Table 3: Cell frequencies of $CD45^+$ frequencies as % of singlets at different time-points shown as mean and standard deviation.

**$CD45^+$ cell frequencies as % of singlets**		
**Time-point**	**Mean**	**Standard deviation**
CTRL	97.08	1.82
Baseline	98.75	1.14
1 -2 months p.s.	98.02	1.94
3 - 5 months p.s.	97.58	1.68
6 - 8 months p.s.	97.29	2.35
9 - 11 months p.s.	93.12	3.30

Suppl. Table 4: Immune cell subsets identified via mass cytometry and corresponding surface markers defining the subsets.

Overview of immune cell subsets and surface markers<br>Major immune cell subsets	
**Subset**	**Markers**
Granulocytes	$CD66b^+$, $CD16^+$, $CD11b^+$
CD4+ T cells	Lin^-, $CD3^+$, $CD4^+$
Cytotoxic T cells	Lin^-, $CD3^+$, $CD8^+$, $NKG2D^+$
NK cells	Lin^-, $CD56^+$, $CD16^+$, $NKG2D^+$
Monocytes	Lin^-, $CD14^+$, $CD64^+$, $CD33^+$
B cells	Lin^-, $CD19^+$
NKT cells	Lin^-, $CD3^+$, $CD56^+$
pDCs	Lin^-, $CD123^+$

CD4+ T cells<br>Lin^-, $CD3^+$, $CD4^+$	
**Subset**	**Markers**
Naïve CD4+ T cells	$CD45RA^+$, $CD197^+$, $CD62L^+$, $CD27^+$
Central memory CD4+ T cells	$CD45RA^-$, $CD197^+$, $CD62L^+$, $CD27^+$
$CD127^+$ central memory subset	$CD45RA^-$, $CD197^+$, $CD62L^+$, $CD27^+$, $CD127^+$
Effector memory CD4+ T cells	$CD45RA^-$, $CD197^-$, $CD62L^-$, $CD27^-$
$CD127^+$ effector memory subset	$CD45RA^-$, $CD197^-$, $CD62L^-$, $CD27^-$, $CD127^+$

NK cells<br>Lin^-, $CD56^+$, $CD16^+$, $NKG2D^+$	
**Subset**	**Markers**
Cytokine producing subset	$CD16^-$, $CD56^{high}$
Cytotoxic subset	$CD16^+$, $CD56^{dim}$

$CD14^+$ Monocytes<br>Lin^-, $CD14^+$, $CD64^+$, $CD33^+$	
**Subset**	**Markers**
Monocytic MDSCs	$HLA\text{-}DR^{low}$
Classical monocytes	$CD14^+$, $CD16^-$, $HLA\text{-}DR^+$
Intermediate monocytes	$CD14^+$, $CD16^+$, $HLA\text{-}DR^+$

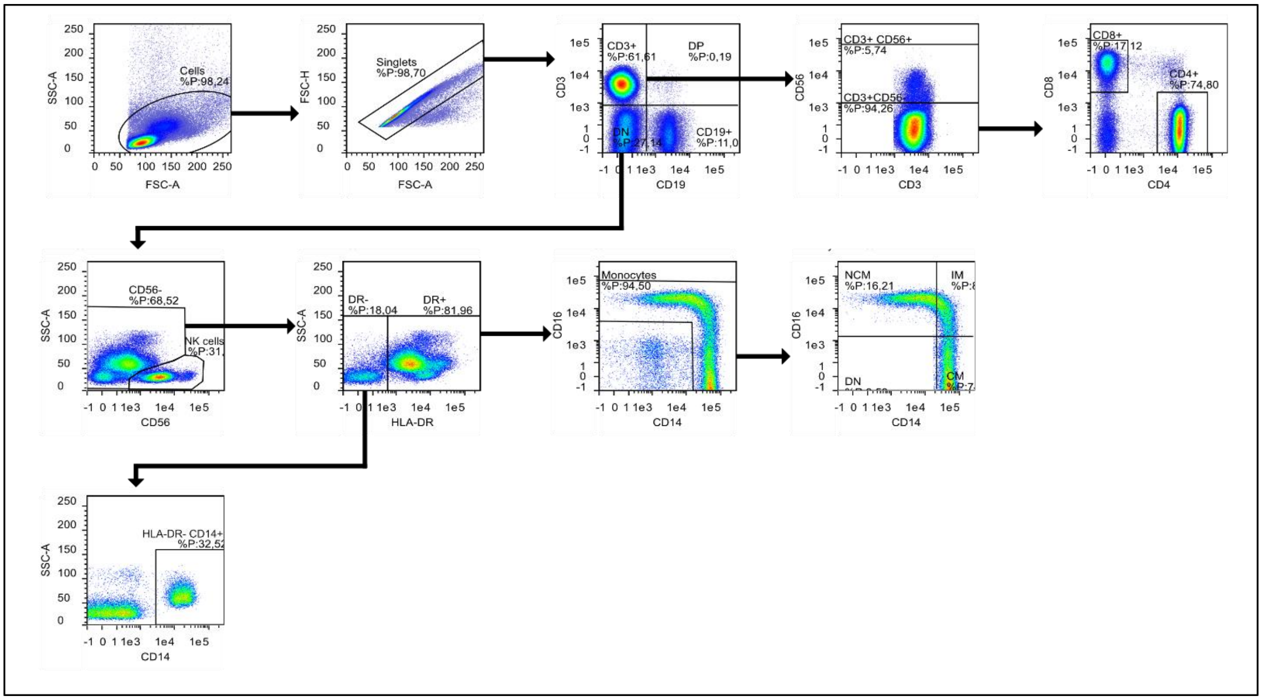

Suppl. Fig. 1: Flow cytometry gating scheme for the identification of $CD3^+$ cells, $CD19^+$ cells, CD4+ cells, CD8+ cells, NK cells, HLA-DR^- $CD14^+$ cells, monocytes, CM, IM and NCM using the markers CD3, CD19, CD4, CD8, CD56, HLA-DR, CD14 and CD16.

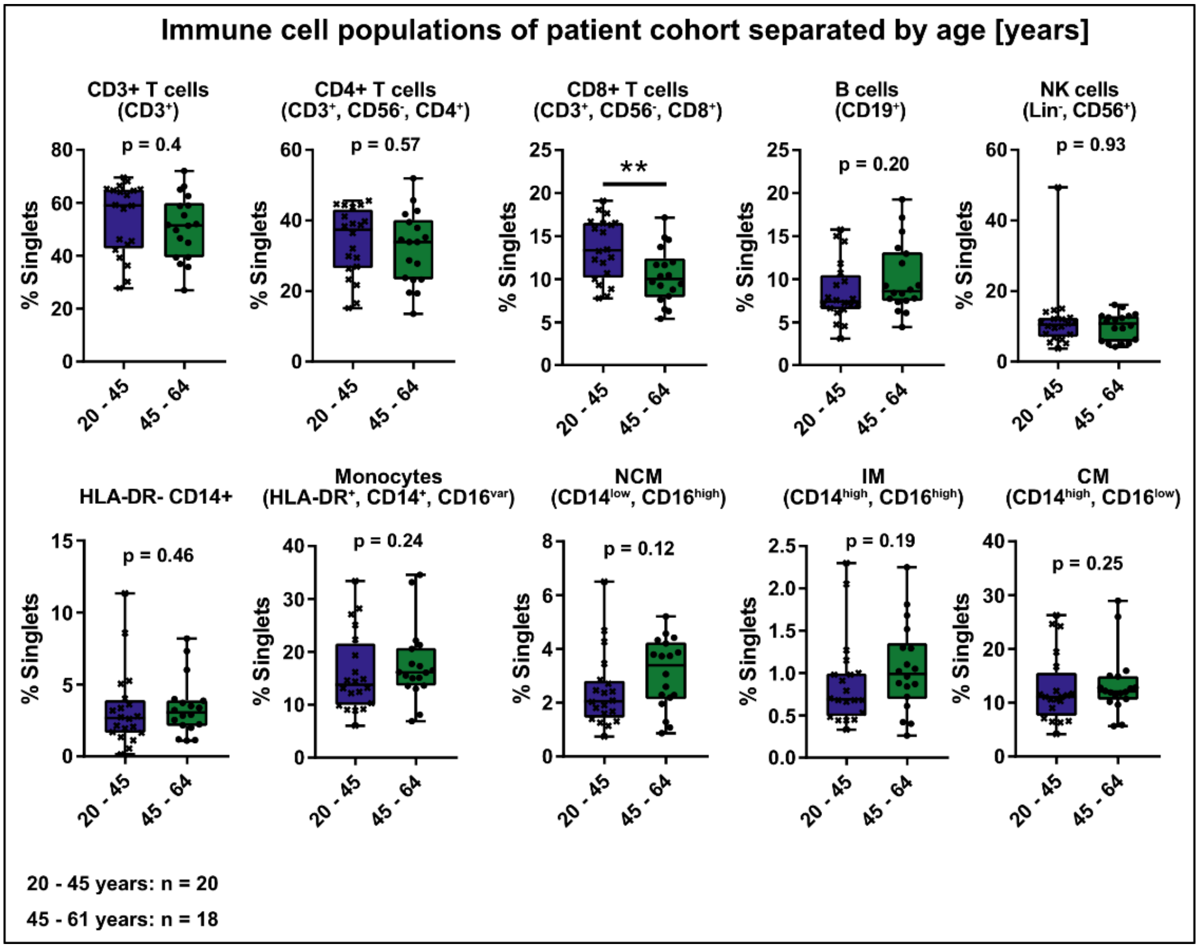

Suppl. Fig. 2: Immune cell populations of the morbid obese patient cohort determined via flow cytometry separated into two groups by age (20 – 45 years and 45 – 64 years). Statistical analysis was performed using unpaired T-test or an unpaired Mann-Whitney test ($\alpha = 0.05$). ** p-value ≤ 0.01.

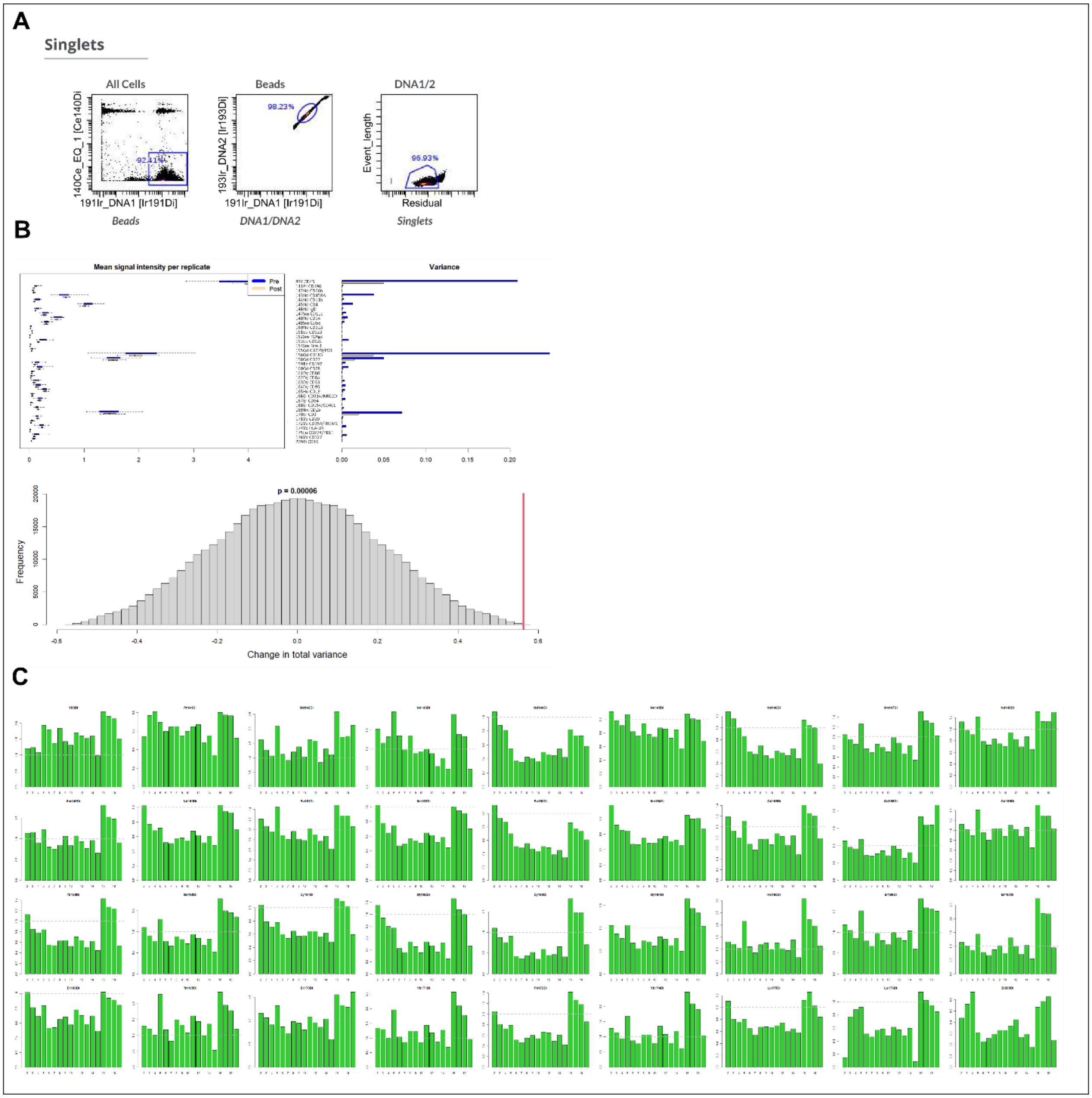

Suppl. Fig. 3: Clean-up of cells (A) and Batch-to-batch normalization using CyTOF Batch Adjust (B + C). FCS files were uploaded into Cytobank and cleaned from beads and doublets (A). Batch-to-batch normalization was performed using CyTOF Batch Adjust resulting in pre- and post-variance plots (B) as well as scaling factors (C).

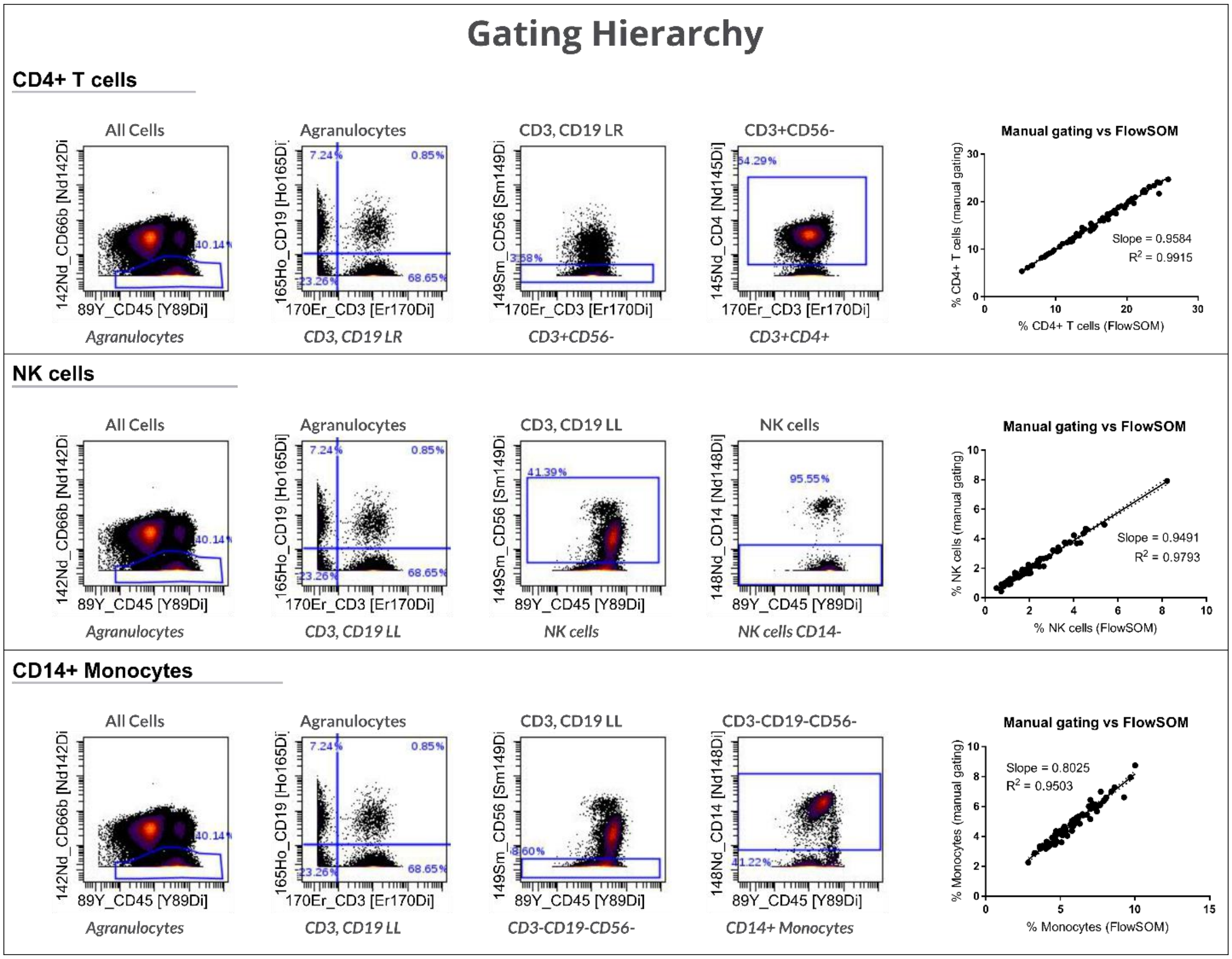

Suppl. Fig. 4: Manual gating scheme of mass cytometry data for CD4+ T cells, NK cells and CD14+ monocytes as well as comparison of manual gating vs. automated clustering using FlowSOM.

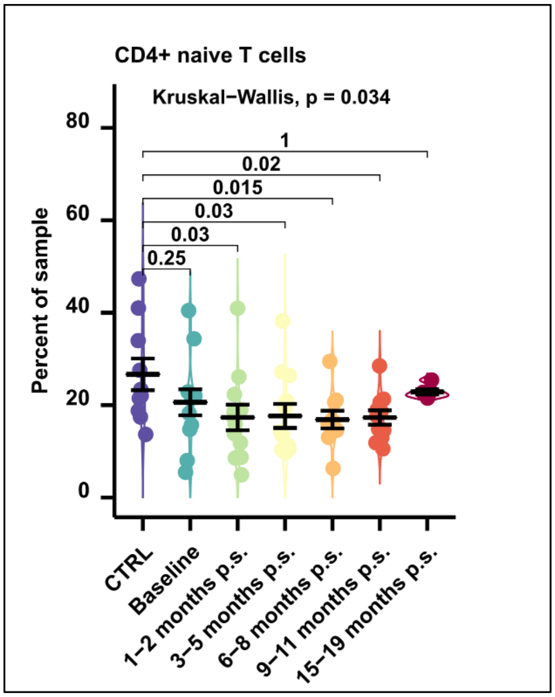

Suppl. Fig. 5: Time-progression of CD4+ naïve T cell subset including the time-points CTRL (n = 10), Baseline, (n = 12), 1 – 2 months p.s. (n = 12), 3 – 5 months p.s. (n = 12), 6 – 8 months p.s. (n = 10), 9 – 11 months p.s. (n = 11), and 15 – 19 months p.s. (n = 6). Statistical analysis was performed using a Kruskal-Wallis test and two sided Wilcoxon Signed Rank Test (α = 0.05) with individual p-values assigned.
