# Supplementary material for: Influence of bariatric surgery on the peripheral blood immune system of female patients with morbid obesity revealed by high-dimensional mass cytometry
Source: Front Immunol. 2023 May 11;14:1131893. doi: 10.3389/fimmu.2023.1131893 (PMC10230950; doi:10.3389/fimmu.2023.1131893)
Supplement: Supplementary file 2 [file DataSheet_2.pdf]

CyTOF Batch Adjust - R Script

```
if (!requireNamespace("BiocManager", quietly = TRUE))
  install.packages("BiocManager")

BiocManager::install("flowCore")

source("BatchAdjust.R")

BatchAdjust(
  basedir=".",
  outdir=".",
  channelsFile = "ChannelsToAdjust.txt",
  batchKeyword="adapt",
  anchorKeyword = "adapt",
  method="99p",
  transformation=FALSE,
  addExt=NULL,
  plotDiagnostics=TRUE)
```

SPECTRE - R Script

```
#######################################################################################
#### Spectre: General Discovery Workflow - (1/4) - Data preparation and
arcsinh transformation
#######################################################################################

# Spectre R package: https://github.com/ImmuneDynamics/Spectre
# Thomas Myles Ashhurst, Felix Marsh-Wakefield, Givanna Putri

#######################################################################################
#### 1. Install Spectre and load packages
#######################################################################################

## Install (if not already installed)
if(!require('devtools')) {install.packages('devtools')}

## Install Spectre
library('devtools')

options(timeout=6000) # this changes the 'timeout' limit for downloading
the package
devtools::install_github("immunedynamics/spectre") # this will download and
install Spectre

## Load spectre
library(Spectre)
library(EnhancedVolcano)
Spectre::package.check()    # Check that all required packages are
installed
Spectre::package.load()     # Load required packages
library(flowCore)

### Set DT threads
getDTthreads()

#######################################################################################
#### 1. Set working directory
#######################################################################################

### Set primary directory
dirname(rstudioapi::getActiveDocumentContext()$path)
setwd(dirname(rstudioapi::getActiveDocumentContext()$path))
getwd()
PrimaryDirectory <- getwd()
PrimaryDirectory

### Set input directory
setwd(PrimaryDirectory)
setwd("data")
InputDirectory <- getwd()
InputDirectory
setwd(PrimaryDirectory)

### Set metadata directory
setwd(PrimaryDirectory)
setwd("metadata")
MetaDirectory <- getwd()
MetaDirectory
setwd(PrimaryDirectory)
```

```
### Set output directory
dir.create("output", showWarnings = FALSE)
setwd("output")
OutputDirectory <- getwd()
setwd(PrimaryDirectory)

#######################################################################################
#### 2. Import and prep data
#######################################################################################
### Import data
setwd(InputDirectory)
list.files(InputDirectory, ".fcs")
data.list <- read.files(file.loc = InputDirectory,
                                 file.type = ".fcs",
                                 do.embed.file.names = TRUE)

### Check the data
check <- do.list.summary(data.list)
check$name.table # Review column names and their subsequent values
check$ncol.check # Review number of columns (features, markers) in each
sample
check$nrow.check # Review number of rows (cells) in each sample
data.list[[1]]

### Merge data
cell.dat <- Spectre::do.merge.files(dat = data.list)
cell.dat

### Read in metadata
setwd(MetaDirectory)
meta.dat <- fread("sample.details.csv")
meta.dat
gc()

#######################################################################################
#### 3. Data transformation
#######################################################################################
setwd(OutputDirectory)
dir.create("Output 1 - transformed plots")
setwd("Output 1 - transformed plots")

### Arcsinh transformation
as.matrix(names(cell.dat))
to.asinh <- names(cell.dat)[c(adapt)]
to.asinh
cofactor <- 5
cell.dat <- do.asinh(cell.dat, to.asinh, cofactor = cofactor)
transformed.cols <- paste0(to.asinh, "_asinh")

#######################################################################################
#### 4. Add metadata and set some preferences
#######################################################################################
### Add metadata to data.table
meta.dat
sample.info <- meta.dat[,c(adapt)] #adapt
sample.info
```

```
cell.dat <- do.add.cols(cell.dat, "FileName", sample.info, "Filename",
rmv.ext = TRUE)
cell.dat

### Columns
as.matrix(names(cell.dat))
cellular.cols <- names(cell.dat)[c(adapt)]
as.matrix(cellular.cols)
as.matrix(names(cell.dat))
cluster.cols <- names(cell.dat)[c(adapt)]
as.matrix(cluster.cols)
exp.name <- "adapt"
sample.col <- "Sample"
group.col <- "Group"
batch.col <- "Batch"
data.frame(table(cell.dat[[group.col]])) # Check number of cells per
sample.
unique(cell.dat[[group.col]])
sub.targets <- c(10000,10000,10000,10000,10000,10000) # target subsample
numbers from each group
sub.targets

#######################################################################################
#### 5. Clustering and dimensionality reduction
#######################################################################################
setwd(OutputDirectory)
dir.create("Output 2 - clustering")
setwd("Output 2 - clustering")

### Clustering
cell.dat <- run.flowsom(cell.dat, cluster.cols, meta.k = adapt)
cell.dat
fwrite(cell.dat, "Clustered.csv")

### Dimensionality reduction
cell.sub <- do.subsample(cell.dat, sub.targets, group.col)
cell.sub
cell.sub <- run.umap(cell.sub, cluster.cols,
                     umap.x.name = "UMAP_X",
                     umap.y.name = "UMAP_Y",
                     umap.seed = 42,
                     neighbours = 30,
                     n_components = 2,
                     metric = "euclidean",
                     n_epochs = 200,
                     input = "data",
                     init = "spectral",
                     min_dist = 0.1,
                     set_op_mix_ratio = 1,
                     local_connectivity = 1,
                     bandwidth = 1,
                     alpha = 1,
                     gamma = 1,
                     negative_sample_rate = 5,
                     a_gradient = NA,
                     b_gradient = NA,
                     spread = 1,
                     transform_state = 42,
```

```
                     knn.repeats = 1,
                     verbose = TRUE,
                     umap_learn_args = NA)
cell.sub
fwrite(cell.sub, "RD.sub.csv")

### Expression heatmap
exp <- do.aggregate(cell.dat, cluster.cols, by = "FlowSOM_metacluster")
exp
make.pheatmap(exp, "FlowSOM_metacluster", cluster.cols, file.name =
"Heatmap_cluster.pdf")

### Make expression plots
make.colour.plot(cell.sub, "UMAP_X", "UMAP_Y", batch.col, col.type =
'factor', filename = "Batch_plot.pdf")
make.colour.plot(cell.sub, "UMAP_X", "UMAP_Y", "FlowSOM_metacluster",
col.type = 'factor', add.label = TRUE, nudge_x = 0, filename =
"Metacluster_overlay.pdf")
make.multi.plot(cell.sub, "UMAP_X", "UMAP_Y", cellular.cols, figure.title =
"Multiplot_cellular")
make.multi.plot(cell.sub, "UMAP_X", "UMAP_Y", cluster.cols, figure.title =
"Multiplot_cluster")
make.multi.plot(cell.sub, "UMAP_X", "UMAP_Y", "FlowSOM_metacluster",
group.col, col.type = 'factor', add.density = TRUE)

########################################################################################
#### 6. Annotate clusters
########################################################################################
setwd(OutputDirectory)
dir.create("Output 3 - annotation")
setwd("Output 3 - annotation")

### Annotate
annots <- list(adapt)
annots <- do.list.switch(annots)
names(annots) <- c("Values", "Population")
setorderv(annots, 'Values')
annots
cell.dat <- do.add.cols(cell.dat, "FlowSOM_metacluster", annots, "Values")
cell.dat
cell.dat <- cell.dat %>% drop_na(Population)
cell.sub <- do.add.cols(cell.sub, "FlowSOM_metacluster", annots, "Values")
cell.sub
cell.sub <- cell.sub %>% drop_na(Population)

### Save data and plots
fwrite(cell.dat, "Annotated.data.csv")
fwrite(cell.sub, "Annotated.data.DR.csv")
make.colour.plot(cell.sub, "UMAP_X", "UMAP_Y", "Population", col.type =
'factor', add.label = TRUE, nudge_y= 0.7, nudge_x = 0,
filename="merged_clusters.pdf")
make.multi.plot(cell.sub, "UMAP_X", "UMAP_Y", "Population", group.col,
col.type = 'factor', add.density = TRUE)
rm(exp)
exp <- do.aggregate(cell.dat, cluster.cols, by = "Population")
make.pheatmap(exp, "Population", cluster.cols, file.name = "Combined
cluster - heatmap.pdf")
```

```
########################################################################################
#### Write summary data
########################################################################################
setwd(OutputDirectory)
dir.create("Output 4 - summary data")
setwd("Output 4 - summary data")

### Select columns to measure MFI
as.matrix(cellular.cols)
dyn.cols <- cellular.cols[c(adapt)]
dyn.cols

### Setup cell count data
as.matrix(unique(cell.dat[[sample.col]]))
meta.dat

# counts <- meta.dat[,c(sample.col, 'Cells per sample'), with = FALSE]
# counts

### Create summary tables
sum.dat <- create.sumtable(dat = cell.dat,
                           sample.col = sample.col,
                           pop.col = "Population",
                           use.cols = dyn.cols,
                           annot.cols = c(group.col, batch.col),
                           #counts = counts
                                            )

as.matrix(names(sum.dat))
sum.dat

### Write summary data
fwrite(sum.dat, 'sum.dat.csv')

########################################################################################
#### Output session info
########################################################################################
setwd(OutputDirectory)
dir.create("Output - info")
setwd("Output - info")

sink(file = "session_info.txt", append=TRUE, split=FALSE, type =
c("output", "message"))
session_info()
sink()
gc()

########################################################################################
#### Analysis session setup
########################################################################################
### Set primary directory
dirname(rstudioapi::getActiveDocumentContext()$path)            # Finds the
directory where this script is located
setwd(dirname(rstudioapi::getActiveDocumentContext()$path))     # Sets the
working directory to where the script is located
getwd()
PrimaryDirectory <- getwd()
```

```
PrimaryDirectory

### Set input directory
setwd(PrimaryDirectory)
setwd("output/Output 4 - summary data")
InputDirectory <- getwd()
InputDirectory
setwd(PrimaryDirectory)

### Set output directory
setwd(PrimaryDirectory)
dir.create("Output 5 - quantitative analysis", showWarnings = FALSE)
setwd("Output 5 - quantitative analysis")
OutputDirectory <- getwd()
setwd(PrimaryDirectory)



#######################################################################################
#### Import data
#######################################################################################
setwd(InputDirectory)

### Read in files
list.files(getwd(), ".csv")
sum.dat <- fread('sum.dat.csv')
as.matrix(names(sum.dat))
sum.dat


#######################################################################################
#### Preferences
#######################################################################################
### Define colums
as.matrix(names(sum.dat))
sample.col <- "Sample"
group.col <- "Group"
batch.col <- "Batch"
annot.cols <- c(group.col, batch.col)
annot.cols
plot.cols <- names(sum.dat)[c(adapt)]
plot.cols

### Comparisons and group order
variance.test <- 'kruskal.test'
pairwise.test <- "wilcox.test"
comparisons <- list(c(adapt))
comparisons
grp.order <- c(adapt)
grp.order

### Sort row order
sum.dat <- do.reorder(sum.dat, group.col, grp.order)
sum.dat[,c(sample.col, annot.cols),with = FALSE]
as.matrix(unique(sum.dat[[group.col]]))

#######################################################################################
#### Stats calculations
```

```
################################################################################
setwd(OutputDirectory)
dir.create("Output 5.1 - summary data tables")
setwd("Output 5.1 - summary data tables")

### Statistical tests
sum.dat.stats.raw <- create.stats(sum.dat,
                                  use.cols = plot.cols,
                                  sample.col = sample.col,
                                  group.col = group.col,
                                  comparisons = comparisons,
                                  corrections = NULL,
                                  variance.test = variance.test,
                                  pairwise.test = pairwise.test)

sum.dat.stats.raw

### Review and save to disk
sum.dat.stats.raw[,c(1:3)]
fwrite(sum.dat.stats.raw, "Summary data - stats - uncorrected.csv")

################################################################################
#### Volcano plots
################################################################################
setwd(OutputDirectory)
dir.create("Output 5.4 - volcano plots")
setwd("Output 5.4 - volcano plots")

### Setup for volcanos
comps <- list()
for(i in c(1:length(comparisons))){
  temp <- comparisons[[i]]
  strg <- paste0(temp[[1]], " to ", temp[[2]])
  comps[[i]] <- strg
}
comps

### Uncorrected volcanos (p-values)
setwd(OutputDirectory)
dir.create("Output 5.4 - volcano plots")
setwd("Output 5.4 - volcano plots")
dir.create("Uncorrected p-values")
setwd("Uncorrected p-values")
for(i in comps){
  temp <- sum.dat.stats.raw[sum.dat.stats.raw[["Comparison"]] == i,]
  p.dat <- temp[temp[["Type"]] == "p-value",]
  p.dat <- p.dat[,names(p.dat)[c(3:length(names(p.dat)))], with = FALSE]
  fc.dat <- temp[temp[["Type"]] == "FClog2",]
  fc.dat <- fc.dat[,names(fc.dat)[c(3:length(names(fc.dat)))], with =
FALSE]
  nms <- names(fc.dat)
  make.volcano.plot(dat.p = p.dat,
                    dat.fc = fc.dat,
                    vars = nms,
                    title = i,
                    xlim = c(-3.5, 3.5),
                    col =c("black","green","blue","red3"))
```

```
}

########################################################################################
#### Output session info
########################################################################################
### Save session info
setwd(OutputDirectory)
dir.create("Output - info")
setwd("Output - info")
sink(file = "session_info.txt", append=TRUE, split=FALSE, type =
c("output", "message"))
session_info()
sink()
```
